# Lung adenocarcinomas without driver genes converge to common adaptive strategies through diverse genetic, epigenetic, and niche construction evolutionary pathways

**DOI:** 10.1007/s12032-024-02344-2

**Published:** 2024-05-05

**Authors:** Robert A. Gatenby, Kimberly A. Luddy, Jamie K. Teer, Anders Berglund, Audrey R. Freischel, Ryan M. Carr, Amanda E. Lam, Kenneth J. Pienta, Sarah R. Amend, Robert H. Austin, Emma U. Hammarlund, John L. Cleveland, Kenneth Y. Tsai, Joel S. Brown

**Affiliations:** 1 Department of Cancer Biology and Evolution, Moffitt Cancer Center, 12902 Magnolia Drive, Tampa, FL 33612 USA; 2 Department of Bioinformatics, Moffitt Cancer Center, Tampa, USA; 3 Cancer Bioengineering Department, University of Texas, Austin, USA; 4 Department of Oncology, Mayo Clinic, Rochester, USA; 5 Case Western Reserve University, Cleveland, USA; 6 Cancer Ecology Program, Johns Hopkins University, Baltimore, USA; 7 Department of Physics, Princeton University, Princeton, USA; 8 Division of Translational Cancer Research, Lund University, Lund, Sweden; 9 Departments of Pathology and Tumor Biology, Moffitt Cancer Center, Tampa, USA

**Keywords:** Cancer evolution, Lung adenocarcinoma, Evolutionary triage, Cancer ecology, Co-adapted syndromes, Driver phenotype

## Abstract

Somatic evolution selects cancer cell phenotypes that maximize survival and proliferation in dynamic environments. Although cancer cells are molecularly heterogeneous, we hypothesized convergent adaptive strategies to common host selection forces can be inferred from patterns of epigenetic and genetic evolutionary selection in similar tumors. We systematically investigated gene mutations and expression changes in lung adenocarcinomas with no common driver genes (*n* = 313). Although 13,461 genes were mutated in at least one sample, only 376 non-synonymous mutations evidenced positive evolutionary selection with conservation of 224 genes, while 1736 and 2430 genes exhibited ≥ two-fold increased and ≥ 50% decreased expression, respectively. Mutations under positive selection are more frequent in genes with significantly altered expression suggesting they often “hardwire” pre-existing epigenetically driven adaptations. Conserved genes averaged 16-fold higher expression in normal lung tissue compared to those with selected mutations demonstrating pathways necessary for both normal cell function and optimal cancer cell fitness. The convergent LUAD phenotype exhibits loss of differentiated functions and cell–cell interactions governing tissue organization. Conservation with increased expression is found in genes associated with cell cycle, DNA repair, p53 pathway, epigenetic modifiers, and glucose metabolism. No canonical driver gene pathways exhibit strong positive selection, but extensive down-regulation of membrane ion channels suggests decreased transmembrane potential may generate persistent proliferative signals. NCD LUADs perform niche construction generating a stiff, immunosuppressive microenvironment through selection of specific collagens and proteases. NCD LUADs evolve to a convergent phenotype through a network of interconnected genetic, epigenetic, and ecological pathways.

**Supplementary Information:**

The online version contains supplementary material available at 10.1007/s12032-024-02344-2.

## Introduction

Evolution by natural selection occurs in populations having heritable phenotypic variation and environmental selection forces that act on births and deaths. Within a given environment, well-adapted phenotypes proliferate at the expense of those that are poorly adapted, and their phenotypic properties are propagated across generations through genetic and epigenetic mechanisms of inheritance. In addition, an organisms’ adaptations can include niche engineering [1] in which they alter their habitat (e.g., a beaver dam or angiogenesis) to locally increase their own fitness. Later generations then inherit this ecological niche. Collectively, these interacting dynamics form “The ecological theater and evolutionary play” [2].

Somatic evolution in cancer cell populations results in phenotypic adaptations to myriad intracellular and extracellular barriers to uncontrolled proliferation. Furthermore, once a cancer population has emerged, it is subjected to heterogeneous intra-tumoral microenvironments producing diverse microenvironmental niches and corresponding adapted cancer cell subpopulations. However, multiple genetic pathways can achieve identical phenotypic properties [3] and this heterogeneity is a fundamental challenge to characterizing somatic evolution and optimizing tumor treatment.

To identify convergent phenotypes and provide insights into common evolutionary selection forces and cellular adaptive strategies, we interrogated mutation and expression data from a cohort of patients (*n* = 313) with lung adenocarcinoma and no common driver genes (NCD LUAD) in the TCGA database.

We applied an approach analogous to identifying environmental selection forces and phenotypic adaptive strategies using molecular data from cave fish [4]. Cave fish live in constant darkness and have a convergent phenotype characterized by loss of eyes [5] and skin pigmentation and gain of function in tactile organs. Over 85 fish species have evolved a cave morph demonstrating multiple genetic pathways [6] can converge to the same phenotype [4]. Observations of the cave fish phenotype readily identify some of their adaptive strategies and, therefore, environmental selection forces. Conversely, analysis of molecular data from a single cave fish provides only limited insights because diverse genetic pathways can lead to the same morphology [7]. However, molecular data from a cohort of fish from different caves will demonstrate common patterns of genetic changes related to eyes, skin, and tactile organs revealing the selection forces (i.e., constant darkness) and adaptive strategies. Additionally, such sampling can produce novel insights, for example, such as the unexpected role of environmental hypoxia in cavefish physiology [8]. Here, we similarly investigate patterns of genetic, epigenetic, and ecological inheritance to infer convergent phenotypic properties and the associated environmental selection forces.

In prior studies [9, 10], we found cancer cells appear to adapt to a relatively fixed number of intracellular and extracellular barriers to unconstrained proliferation [10]. Driver genes function by simultaneously overcoming many barriers through their interactomes. In contrast, cancer without driver genes must adapt to each barrier more or less one at a time. Thus, we hypothesized maximum insight into convergent adaptive strategies [11] would be obtained from cohorts of cancers without driver genes that evolved in the same tissue environment.

Here, we demonstrate NCD LUADs converge to a phenotype that loses differentiated functions of normal lung epithelial cells, including gas exchange and circadian entrainment, as well as membrane proteins related to cellular interactions with other cells and the extracellular matrix that govern 3-dimensional tissue organization. Like the eyes of a cavefish, these functions incur a cost and provide no fitness benefit to a cancer cell. Across patients, consistent selection of specific collagen and protease family demonstrates that appear to direct niche engineering of stiff, immunosuppressive microenvironments. The convergent phenotype conserves and upregulates genes related to cell cycle, mitosis, the p53 pathway, and epigenetic modification. Although no canonical driver gene pathways exhibit strong selection, we find striking down-regulation of membrane ion channels suggesting loss of the transmembrane potential may generate a driver phenotype that promotes unconstrained proliferation. We find a complex but consistent selection on elements of adaptive immunity, inflammation, and antigen presentation suggesting potential strategies for therapeutic intervention in this class of LUAD.

## Methods

### Gene list acquisition

We divided the TCGA lung adenocarcinoma cohort based on known driver mutations in *KRAS* (G12, G13, Q61, A146), *BRAF* (V600, N581, G464, G466, G469, G596, D594), and *EGFR* (L858, S768, L861, G719, T790, indels in exons 18–21). The 313 non-EGFR/KRAS/BRAF patients were classified as no common driver (NCD). Somatic mutations were downloaded from the TCGA PanCancer Atlas [12]. Tumor and normal sequence alignment files used to calculate individual base coverage were downloaded from Genome Data Commons. A base was considered sufficiently covered if the depth of coverage was ≥ 14 in tumor sample and ≥ 8 in normal samples as has been previously described: https://www.synapse.org/#!Synapse:syn1695394. The fraction of each gene’s protein coding bases (using the longest RefSeq transcript) covered by sufficient sequence data was calculated for each sample using the Negative Storage Model [13]. Gene-level depth of coverage was then determined by calculating the number of bases sufficiently covered by sequencing for each of the RefSeq coding genes (with 25 base-pair flanking regions). This procedure measures the fraction of each gene (longest transcript) covered by sequencing data. To address sequencing artifacts that falsely decrease mutation rates, we excluded genes with low average depth of coverage frequency (< 50%) and those with errors in the RefSeq gene model.

TCGA data is whole exome sequencing with paired tumor/normal analysis to exclude germ line mutations. The identified mutations include non-synonymous, synonymous, intronic, UnTRanslated region, and intergenic. For our analysis, we limit the mutations to protein altering mutations: non-synonymous and truncating (stop-gain, frameshifting indel, splicing).

To minimize potential artifacts related to expression and to focus on genes that are likely functional, we examined differential gene expression between tumor samples and normal adjacent tissue from each patient. Genes were included only if their expression (log base 2) was ≥ 1.5 in either cancer or normal tissue. In each gene, the average expression in normal tissue and tumor samples across the cohort was analyzed separately to supplement the genetic data.

### Mutational frequency

**Fig. 1:**
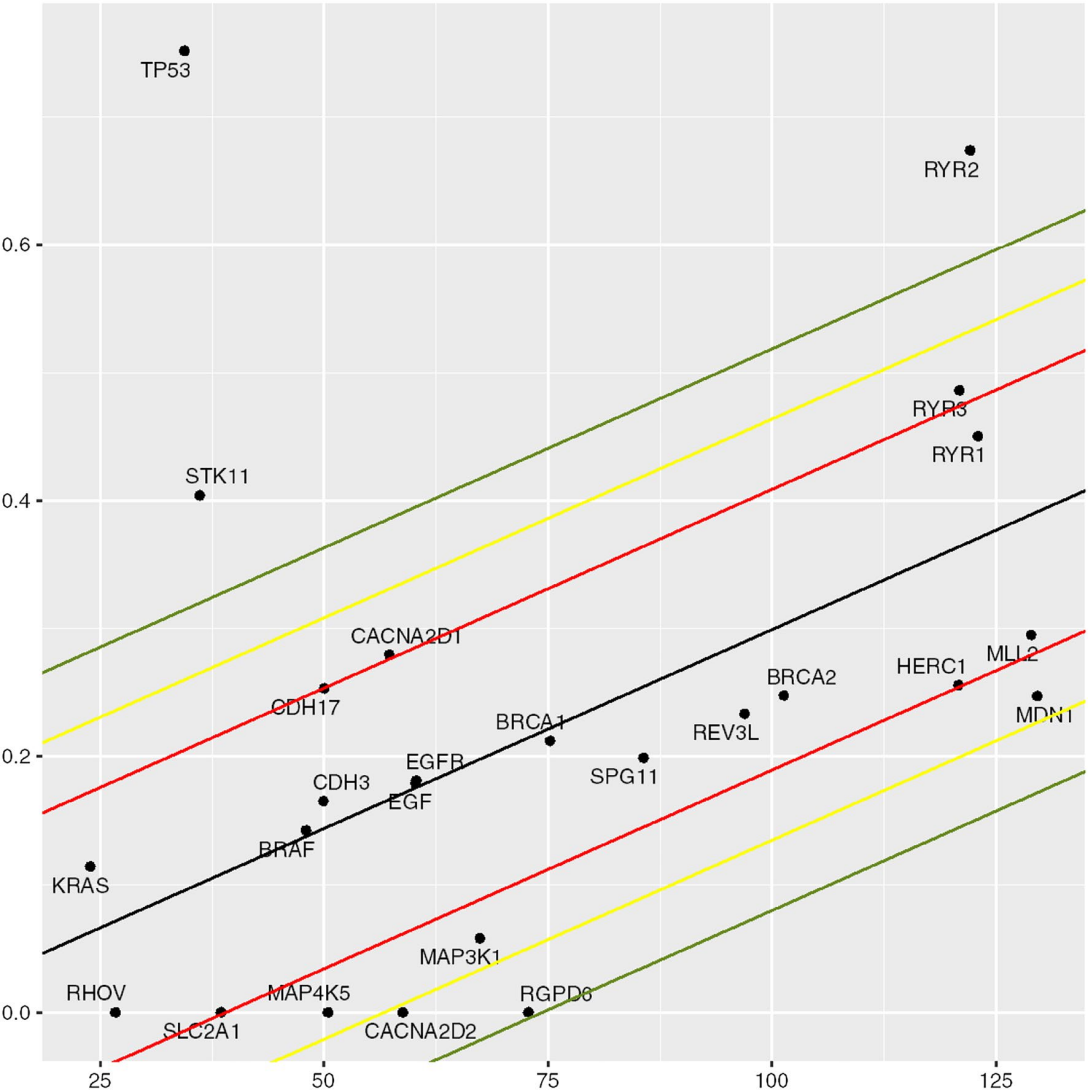
Converting gene mutation number into evolutionary selection. The number of mutations within the cohort are plotted against the gene size (number of base pairs). Using linear regression, a neutral line was established for an expected number of mutations for each gene size with no evolutionary selection (i.e., “passenger mutations”). When the number of mutations exceeded 2 standard deviations above the neutral line, the mutation was considered under positive evolutionary selection. Genes in which the number of mutations was 2 standard deviations below the neutral line were considered “conserved.”

Our approach identifies genes in NCD LUADs that are mutated more or less frequently than expected based on chance. To identify over- and under-mutated genes, we plotted the observed frequency of samples with non-synonymous mutations in each gene against that gene’s size (number of base pairs). Assuming the probability of mutation was approximately equal for every base pair in an expressed gene, the background mutation rate was determined by regressing the mutational frequency of each gene against gene size (Fig. 1). The distance of each gene to the regression line was then determined, and this standardized residual was compared across all genes. Negative and positive residual values indicate under-mutated and over-mutated genes, respectively. We scored a gene as over-mutated and under positive selection if it was ≥ 2 standard deviations above the neutral line. Similarly, a gene was considered under-mutated (i.e., conserved) and under stabilizing selection if it was ≥ 2 standard deviations below the neural line. In other words, our primary metric for natural selection was based on whether mutations to a gene were less (stabilizing selection) or more (directional selection) frequent than expected by chance.

Our approach is similar to prior studies identifying “essential genes” in bacterial [14] and human genomes [15]. We note, however, that essential genes will be context dependent. Genes necessary for cancer cell proliferation in the host may differ significantly from genes required for normal tissue function. Furthermore, selection on genes for in vivo cancer cells inhabiting the host will differ from those selected for in vitro cancer cells growing in culture media.

We note that our methodology for identifying evolutionary selection of gene mutations assumes a roughly equal probability for mutations in all base pairs and, therefore, differs from prior studies that find variation in mutation rates related to gene expression and chromosomal location [16]. However, as discussed in prior publications [9, 10], variation in the observed mutation rates of individual genes resulting from decreased fitness with loss of the cell due to evolutionary selection could be interpreted as the result of intra-genomic variation in mutation rates. For example, Monroe et al. [17], observed that, in *Arabidopsis thaliana* “genes subject to stronger purifying selection have a lower mutation rate.” In our hypothesis, important regions of the genome are *observed* to be mutated less often [18] because such mutations reduce fitness and proliferation and, therefore, not transmitted across generations. Furthermore, the retention or loss of a gene mutation via natural selection (even if the underlying mutation rate is different) fundamentally depends on its contribution to fitness. Finally, a prior study that reported higher expression levels in over-mutated genes was based on comparisons of expression from cell lines to mutation data from clinical samples [16], which represent very different environmental selection forces. In contrast, here we use expression and mutation data from the same clinical source and find the expression of conserved genes is, on average, 16-fold higher in normal tissue compared to normal tissue expression in genes found to have selected mutations in the cancer cells.

### Identifying conserved pathways and functions

**Fig. 2:**
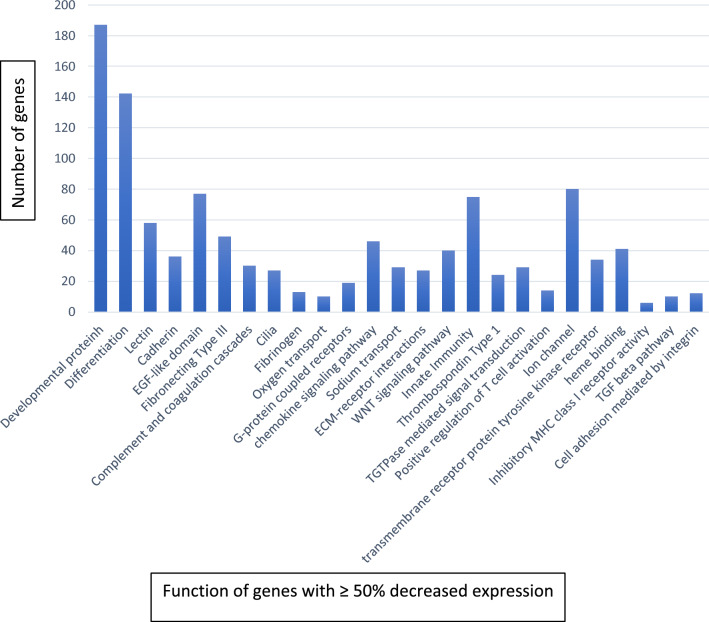
Evolutionary contribution of genes with upregulated expression. Functional clustering of genes with ≥ 50% decreased expression in NCD LUADs compared to normal lung tissue using the DAVID Bioinformatic tool. All listed functions are *p* < 0.05

**Fig. 3:**
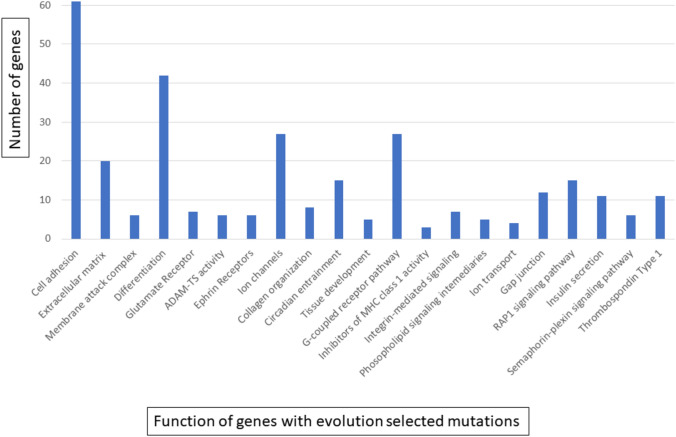
Phenotypic consequences of genes with evolutionarily selected mutations (see Fig. 1). Functional annotations of gene mutations under evolutionary selection (≥ 2 standard deviations above the neutral line in Fig. 1) using DAVID Bioinformatics tool with *p* < 0.05

**Fig. 4:**
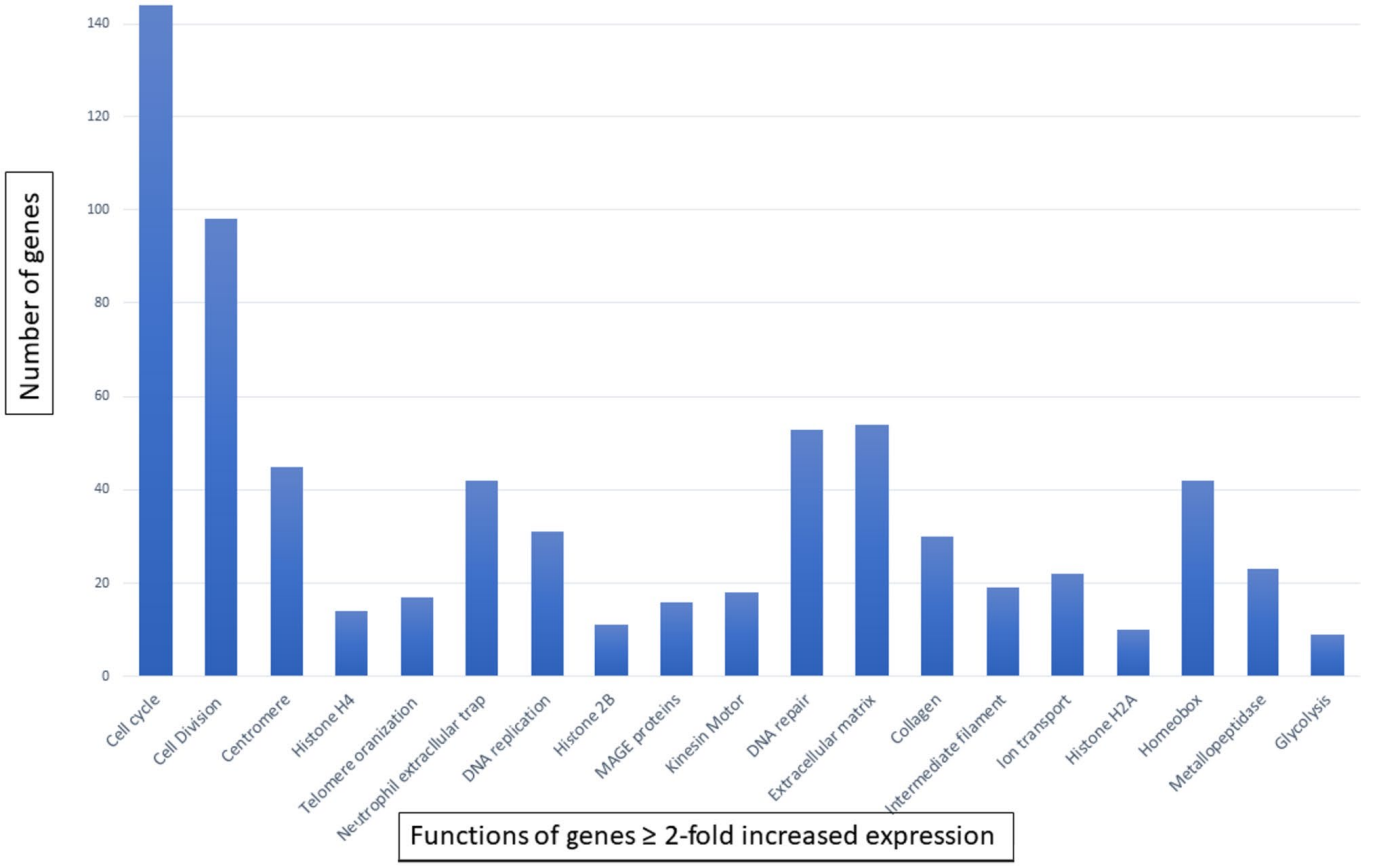
Phenotypic properties of genes with increased expression in NCD LUAD compared to normal lung Functional annotation clustering of genes with ≥ two-fold increased expression compared to normal tissue using DAVID Bioinformatic Tool with *p* < 0.05

To determine coordinated functions of related groups of genes, curated lists of selected or conserved genes were subjected to pathway analysis using DAVID (Database for Annotation and Integrated Discovery), available at the website https://david.ncifcrf.gov. Both Gene Ontology (GO)-DIRECT and -FAT were used to identify significant ontologies, including Biological Process (BP), Cell Compartment (CC), and Molecular Function (MF). We then performed functional annotation clustering (Figs. 1, 2, 3, 4). We selected clusters based on high numbers of queried genes and biological significance. *p* values are from the DAVID tool and represent the probability that the observed cluster could be the result of chance alone. Venn diagrams were constructed using the Ghent University VIB/UGent Center website: http://bioinformatics.psb.ugent.be/webtools/Venn/.

## Results

### Mutational and expression features of NCD lung adenocarcinoma

The initial dataset consisted of 17,112 genes. The mutational but not expression component of the dataset has been previously investigated [9, 10]. Of these, 15,487 genes met our expression-level criterion for inclusion. Of this subset, 13,461 genes were mutated in at least one sample. Using the methodology described above, we identified 388 genes that met criteria as over-mutated (under directional selection, ≥ 2 s.d. above the regression [neutral] line), 264 genes that were conserved and under-mutated (under stabilizing selection, ≤ 2 s.d. below neutral line), and, as would be expected, 11,790 genes fell within 1 s.d. of the neutral line (Table 1). 1736 and 2431 genes demonstrated ≥ two-fold increased or ≥ 50% decreased expression compared to normal tissue, respectively. Of these genes, 556 and 918 demonstrated ≥ four-fold increase or ≥ 75% decreased expression, respectively. 8345 genes had changes in expression between tumor and normal tissue between 50% higher or 33% lower (Table 2).

**Table 1:** Evolution selection for gene mutations and conservation

	Genes evolutionarily conserved (≥ 2 SD below neutral line)	Genes with expected number of mutations (within 1 SD of the neutral line)	Genes with evolutionary selection for mutation (≥ 2 SD above neutral line)
Number of genes	264	11,790	388
Average expression in normal lung (log base 2)	9.00 ± 0.262	8.06 ± .056	5.12 ± 0.312
Average expression in tumor (log base 2)	9.10 ± 0.224	8.03 ± .054	4.46 ± 0.282
Decreased expression in tumor cell by ≥ 50%	19 (7.2%)	1720 (14.6%);	164 (42.3%)
Increased expression in tumor cells by ≥ two-fold	25 (9.5%)	1309 (11.1%)	39 (10.0%)

Evolutionary selection for conservation is more common in genes that are highly expressed in normal tissue with selection for mutations in genes that, on average, had about 16-fold decreased expression in normal tissue

**Table 2:** Comparing epigenetic selection (changes in expression) with genetic selection (mutational changes)

	Genes with > 50% decreased expression in lung cancer	Genes with > two-fold increased expression in lung cancer	Genes with minimal change in expression In lung cancer	Genes with > 75% decreased expression in lung cancer	Genes with > four-fold increased expression in lung cancer
Number	2430	1736	936	917	556
Total mutations	17,300	8905	3665	7002	2971
Average number of mutations per gene	7.1	5.1	3.9	7.6	5.3
Evolutionarily conserved	19	21	19	6	10
Evolution selected mutated genes	163	47	6	63	12

Conserved genes (*n* = 264) in the NCD LUADS had significantly greater expression in normal lung compared to than mutated genes (*n* = 11,790, between 1 s.d. of neutral line) which had significantly greater expression than highly mutated genes (*n* = 388) (Table 1): 9.00 > 8.06 > 5.12, respectively (1-way ANOVA, *F*_2,12439_ = 197.2, *p* < 0.001, with all 3 pairwise comparisons *p* < 0.001 with Bonferroni correction. All expression values are log_2_). Thus, Genes that are conserved in NCD LUADs have an almost 16-fold greater expression in normal lung tissue compared to genes with evolutionarily selected mutations (Tables 1 and 2) suggesting their function is vital to both normal cell functioning and optimizing cancer cells’ fitness.

Similarly, expression of conserved genes in the tumor is greater than neutrally mutated genes which is greater than highly mutated genes: 9.10 > 8.03 > 4.46, respectively (1-way ANOVA, *F*_2,12439_ = 306.9, *p* < 0.001, with all 3 pairwise comparisons *p* < 0.001 with Bonferroni correction). Note the divergence in expression of conserved and highly mutated genes are even greater in the cancer cells than in the normal tissue. Finally, using a paired t test to compare expression levels between tumor and normal tissue, we find significantly increased expression in highly mutated genes (*t*_387_ = 8.70, *p* < 0.001) and neutrally mutated genes (*t*_11789_ = 3.00, *p* < 0.003), and no significant difference in conserved genes (*t*_263_ = 1.81, *p* = 0.07).

Consistent with these results, when compared to normal tissue, only 25 and 19 conserved genes had ≥ two-fold increase and ≥ 50% decrease in expression, respectively; but, among the highly mutated genes, 39 and 164 had ≥ two-fold increase and ≥ 50% decrease in expression, respectively (*χ*^2^ = 26.64, df = 1, *p* < 0.001 for 2 × 2 table of conserved versus highly mutated by ≥ two-fold increase versus and ≥ 50% decrease in expression). The pattern continues to hold for more extreme changes in gene expression. When compared to normal tissue, only 10 and 6 conserved genes had ≥ four-fold increase and ≥ 75% decrease in expression, respectively; but, among the highly mutated genes, 13 and 64 had ≥ four-fold increase and ≥ 75% decrease in expression, respectively (*χ*^2^ = 14.81, df = 1, *p* < 0.001 for 2 × 2 table of conserved versus highly mutated by ≥ four-fold increase versus and ≥ 75% decrease in expression).

Thus, the general pattern suggests many gene mutations in NCD LUADs are loss of function in genes that otherwise have decreased expression or gain of function mutations in genes that otherwise have increased expression. A smaller number of mutations may alter the function of a gene (i.e., “repurpose it”) resulting in a variable change in expression. Similarly, some multifunctional genes may be conserved primarily for a single function resulting in decreased expression.

As an example of these dynamics, we find *STK11,* a multifunctional gene [19] that acts as a tumor suppressor in non-small cell lung cancers [20], is highly mutated and its expression is reduced by about 50%. Mutations in *STK11* in lung cancer are truncating and truncation with loss of function [21]. This association of loss of function mutations in a gene with decreased expression represents “hard wiring” of a gene that, respectively, required increased or decreased expression for optimal fitness.

### Convergent adaptive strategies: loss of differentiated tissue functions

#### Down-regulation of gas exchange and loss of cell adhesion that organize cells in lung tissue

Lung epithelial cells promote gas exchange but, in an evolving cancer cell, these functions, similar to eyes in a cavefish, require resources that provide no fitness benefit and are, thus, eliminated to redistribute resources to function required to survive and proliferate in the tumor microenvironment. This is clearly evident in NCD LUAD, where there is a striking and uniform decreased expression of all genes associated with gas exchange and other differentiated functions.

Genes with ≥ 50% decreased expression (Fig. 2) are highly enriched for gaseous exchange (*p* = 1.0E−5) and carbon dioxide transport (*p* = 4.1E−7), as well as for lectin (*p* = − 1.3E−9), ion transport (*p* = 1.2E−6), thrombospondin (*p* = 7.2E−9), cilia movement (*p* = 3.0E−8), developmental proteins (*p* = 1.5E−3), scavenger receptor (*p* = 4.7E−6), water transmembrane transport (*p* = 9.1E−5), and positive regulation of immune response (*p* = 9.2E−4).

Normal lung tissue maintains a specific 3-dimensional morphology required for organ function, but again genes required for tissue architecture provide no evolutionary benefit to individual cancer cells and, in fact, probably inhibit carcinogenesis. In accord with this notion, there is > 50% expression reduction in multiple genes related to cell–cell interactions, including cadherins, protocadherins, G-protein-coupled receptors, integrins, semaphorins, BMP, and neural adhesion gene families (Fig. 2). Normal lung tissue maintains a specific 3-dimensional morphology, but this also provides no evolutionary benefit to individual cancer cells and, in fact, probably inhibits carcinogenesis. Thus, we find > 50% expression reduction in multiple genes related to cell–cell interactions, including cadherins, protocadherins, G Protein-coupled receptors, integrin, and semaphorins, BMP, and neural adhesion gene families.

#### Loss of ion channels

Genes associated with membrane ion channels are highly mutated with decreased expression suggesting a loss of functions in NCD LUAD (Figs. 2 and 3 and Supplemental Table 1). Such genes influence osmotic regulation [22] and maintain transmembrane ion gradients that regulate a broad range of cell and tissue functions [23]. Notably, there is generally down-regulation of genes encoding K + channels, which are the main contributor to the transmembrane potential [24], and Ca^++^ channels that are extensively involved in intracellular signaling [25]. In addition, we find strong selection for mutations and decreased expression in cholinergic, glutamate, and GABA receptors associated with ion fluxes as well as synaptic regulators [26]. Expression of *SLC6A4*, member of a family of serotonin transporters related to Na^+^ and pH dynamics [27], shows > 99% decreased expression compared to normal lung cells while other family members, *SLC6A13* (a GABA transporter) and *SLC6A20* (a sodium: neurotransmitter symporter*)* show 75–90% decreased expression.

### Niche construction

#### Remodeling of the extracellular matrix (ECM)

Expression data also revealed that NCD LUADs engage in extensive ECM (Supplemental Table 2) remodeling with up to 64-fold increased expression of specific collagen genes (*COL10A1, COL11A1, COL1A1, COL22A1, COL11A2, COL3A1, COL24A1, COL5A1, COL5A2, COL7A1)* and up to 90% decreased expression in other collagen family members (*COL13A1, COL21A1, COL4A3, COL4A4, COL4A6, COL6A6)*. Similarly, some of the most highly mutated genes in NCD LUAD include *COL11A1, COL12A1, COLK3A1, COL5A2, COL6A3, COL6A6, COL14A1, COL19A1,* and *COL22A1*.

The related fibrillin (FBN) and latent transforming growth factor β (LTPB) families [28] are under strong selection for mutations and changes in expression, with *FBN2* and *LTPB1* among the most highly mutated genes and there are marked reductions in the expression of *FBN3*(90%) and *FBLN1, FBLN5,* and *LTBP2* (50–75%).

Similarly extensive evolutionary changes are seen in the ADAMTS gene family of metalloproteinases that direct collagen fibril formation. For example, *ADAMTS16, ADAMTS18,* and *ADAMTS14* are highly mutated and exhibit > four-fold increased expression whereas *ADAMTS8* shows > 80% decreased expression and *ADMTSL3* and *ADAMTSL4* show 75–90% decreased expression. In contrast, matrix metallopeptidase genes *MMP1, MMP10, MMP11, MMP13, MMP3,* and *MMP9* are uniformly increased in expression by 4- to 32-fold. Finally, there are high mutation rates and decreased expression in the mechanosensitive ryanodine receptors (*RYR1, RYR2, RYR3*). In addition, *COL10A1* and *COL11A1* (increased expression of 25- and 64-fold, respectively) are associated with altered immune landscapes and increased stiffness [29, 30].

#### Angiogenesis and vascular maturation

Angiogenesis is a critical component of tumor niche construction. Consistent with this, *VEGFA* and *VEGFB* are conserved (Supplemental Table 2). Both genes have a high level of expression in normal lung cells, which remains unchanged suggesting the continuous vascular remodeling in tumors is comparable to that found in normal lung tissue. In contrast, expression of *VEGFD,* implicated in angiogenesis and lymphangiogenesis [31]*,* has > 90% reduced expression. Similarly, consistent with the chaotic vascular structure of tumors, there are marked decreases in expression of genes associated with vascular maturation, including multiple members of the angiopoietin and angiopoietin-like families (*ANGPT1, ANGPT4, ANGPTL1, ANGPTL5, ANGPTL7)* suggesting this family of genes is essential for normal lung tissue function but does not confer a fitness advantage in cancer cells.

#### Promoting extracellular acidosis

Consistent with the Warburg effect, there is a general shift of NCD LUAD metabolism toward adaptations for hypoxia and acidosis. Unlike normal lung cells, glycolysis genes are highly represented among the 100 highest expressed genes (*p* = 3.6E−3) in NCD LUAD (Supplemental Fig. 1). *HIF1A* is highly conserved with increased expression, while *HIF2A* (now *EPAS1*) and *HIF3A* have > 75% decreased expression. In line with the cancer cells’ increased uptake of resources, *SLC2A1* and *SLC2A5* (glucose transporters *GLUT1* and *GLUT 5*) show an eight-fold increased expression, while other members of the SLC2A family (*SLC2A12, SLC2A14, SLC2A3*) show > 50% decreased expression Finally, while four members of the carbonic anhydrase family (*CA1, CA2, CA3, CA4*) show > 75% decreased expression, the expression of *CA9*, which promotes acid tolerance and immunosuppression [32, 33], is increased 32-fold.

#### Changing immune interactions

Among the most highly expressed genes in normal lung cells (Supplemental Fig. 1) are those that relate to innate immunity to respond to inhaled infectious agents and antigen processing and presentation, to prevent auto-immune responses.

In NCD LUADs, we find a general pattern (Supplemental Table 3) of increased mutations and decreased expression of genes associated with innate immunity (*LILRA1. LILRA2, LILRA3, LILRA5, LILRA6, LILRB1, LILRB2, LILRB3, MARCO, C6, C7, C8, CFP*) and components of the membrane attack complex superfamilies (*BRINP2, BRINP3, ASTN1, ASTN2*) that are also associated with neural development [34, 35].

There are mixed changes in expression of interleukin-related genes in NCD LUAD that could reflect changes in cancer cell production or in the activities of infiltrating host immune cells. For example, there are 50% decreased expression in *IL1, IL1R1, IL1RL1, IL5, IL6, IL11, IL16, IL17D, IL17RA, IL17RE, IL18, IL20RA, and IL34*, but > two-fold increased expression in IL1R2, IL2, IL17C, IL17RD, IL17REL, *IL20, IL20RB, IL23, IL2, IL31RA, IL36, IL37,* and *IL4I1*.

Expression of genes related to antigen processing (*AGER, HLA-E, HLA-DPA1, HLA-DRB1, EPAS1*) and innate immunity (*HLA-E, SLP1, SERPING1, SFTPD, COLEC10, COLEC12*) show 50 to 80% decreased expression. Interestingly, *ERAP1*, responsible for trimming intracellular proteins for presentation on the MHC1 complex [36] and limiting presentation of unstable and immunogenic peptides [37], is also conserved.

In accord with the selection for evolving immune evasion, checkpoint-related genes *PDCD1, PDCD2L, PDCD6, LAG3*, and *CTLA4* are generally conserved and show increases in their expression (two- to three-fold) in NCD LUAD. In contrast, expression of *CD274* (*PD-L1*), which may reflect changes in the immune infiltrate, is decreased by 60%.

While CD markers are generally seen as associated with immune-related cells, they are also expressed on cancer and normal cells [38]. Overall, 29 members of the CD family had at least 50% decreased expression in NCD LUADs (including 16 of the 30 most highly expressed CD genes in normal lung tissue). Only 3 CD genes (*CD19, CD79A, CD27*) had a > two-fold increased expression in NCD LUADs, probably reflecting changes in the immune infiltrates [39, 40]. Of note, there are marked declines of expression in all members of the CD300 family, which is associated with inflammation [41]. Noteworthy, CD300A overexpression has been found to inhibit progression of NSCLC [42] and decreased expression of CD300LG (> 90% expression decrease) has been reported in lung cancers [43].

#### Canonical oncogenic pathways

By definition, NCD LUADs lack conventional driver genes. We thus investigated the mutational and expression changes in a broad range of canonical oncogenic pathways. *TP53* is the most mutated gene in the cohort and notably 11 genes in the P53 signaling pathway (*GTSE1, CHEK1, CCNB1, CCNB2, CCNE1, CCNE2, CDK1, CDKN2A, CDKN3, IGFBP3, RRM2, SERPINB5*) have > four-fold increased expression vs, normal lung tissue (Supplemental Table 4). We also note > two-fold increased expression in some members of the Ephrin pathway (*EPHA10, EPHB1, EPHB2, EPHB3*) that may play key roles in tumor progression, invasion, and immune evasion [44–47].

Though we recognize that signaling circuits are often regulated by post-translational modifications, it is noteworthy that similar patterns of increased expression of other canonical oncogenic pathways are lacking in NCD LUAD. In the MAPK pathway, for example, only EGF shows a > two-fold increased expression, while *MAP3K15, MAP3K3, MAP3K8, MAPK10*, and *MAPK4* show 50 to 75% decreased expression. In contrast, there is conservation of *MAP3K1* and *MAP4K5,* which may promote pro-tumor inflammatory response [48].

Similarly, while *WNT10A* shows a two-fold increased expression, other WNT ligands such as *WNT11, WNT2, WNT2B, WNT3A, WNT7A*, and *WNT9A* show > 50% decreased expression in NCD LUAD. Finally, patterns of stable or decreased expression are seen in genes belonging to canonical signaling pathways: SMAD, NOTCH, PIK3, RB, MYC, HIPPO, AKT, ERBB, and TGF pathways (Supplemental Table 4).

### Essential cellular functions for malignant growth

**Fig. 5:**
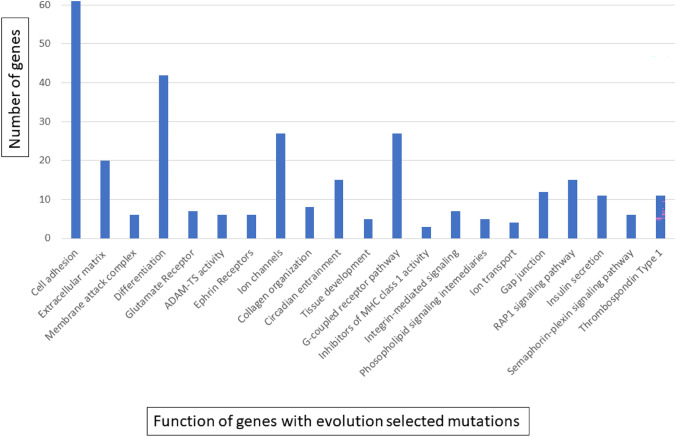
Cellular functions associated with evolutionarily conserved genes (see Fig. 1). Functional annotation clustering of genes that are conserved (≥ 2 standard deviations below the neutral line) using DAVID Bioinformatics Tool with *p* < 0.05

Notably, conservation and increased expression in genes associated with cell cycle, mitosis, and translation (Figs. 4 and 5). For example, genes with > four-fold increased expression were enriched for cell cycle (*p* = 4.3E−32), mitosis (*p* = 3.7E−30), DNA unwinding (*p* = 2.5E−9), DNA replication (*p* = 6.7E−8), centromere function (*p* = 2.2E−17), P53 signaling pathway (*p* = 1.8E−5), and DNA damage repair (*p* = 1.7E−8). Members of the phospholipase A family (*PLA1A, PLA2G12B, PLA2G2A*), which inhibit proliferation and migration in LUAD [49], show 50 to 75% decreased expression. These findings are all consistent with cancer cells evolving increased capacity for proliferation.

We find general upregulation of genes associated with epigenetic mechanisms of modification and evolution (Supplemental Table 5). For example, 6 histone genes (*HIST1H2AE, HIST1H2AM, HIST1H2BD, HIST1H2BG, HIST1H3D*) have > four-fold increased expression. Genes with > four-fold increased expression are enriched for homeobox (*p* = 1.4E−4), translation (*p* = 1.4E−5), cellular senescence (*p* = 7.9E−4), and RNA Polymerase II (*p* = 4.8E−3) functions. These are consistent with cancer cells evolving increased metabolic scope and phenotypic/epigenetic plasticity. Interestingly, there are opposite trends regarding selection on mRNA editing genes. Interestingly, there is mixed selection on mRNA editing genes with significant reductions in the expression of *APOBEC3A* and *APOBEC4* while *APOBEC3B* is conserved (0 mutation in the cohort) with > four-fold increased expression and *APOBEC1* has three-fold increased expression. The divergent expression of *APOBEC3A* and *APOBEC3B* suggests differential fitness contributions of their distinct downstream signatures [50].

Regarding translation, 15 ribosome genes are among the top 100 most highly expressed genes in normal lung and NCD LUADs. However, the cancer cells add 10 other ribosome genes in their top 100. While this increase is consistent with more rapid proliferation, the consistent upregulation of specific ribosome components (from a total of 73 [51]) suggests they may play non-canonical roles in driving and sustaining the malignant state.

## Discussion

Emergence of cancer cell from normal tissue requires an evolutionary transition from a cellular state in which fitness is defined by that of the multicellular organism to one that is self-defined so that its survival and proliferation is independent of host control and governed by its interactions with local environmental selection forces [52, 53]. Somatic evolution requires these phenotypic changes to be heritable via genetic, epigenetic, or ecological (niche construction) mechanisms.

We investigated the Darwinian dynamics of lung adenocarcinomas with no common driver genes prior to clinical therapy through observed mutations and expression data of a large cohort. While some uniqueness in the evolutionary arc is assumed for every NCD LUAD, we predicted consistent patterns of genetic and epigenetic changes among members of the cohort. Indeed, such patterns were manifest and revealed common phenotypic properties of this lung cancer subtype that maximize their fitness.

Although current models of carcinogenesis are built upon the sequence of genetic mutations, we find epigenetic changes in gene expression quantitatively exceed the effects of gene mutations. We find a clear relationship between epigenetic and genetic inheritance as genes with evolution selected mutations frequently also show significant increased or decreased expression. The findings suggest an evolutionary equivalency and that epigenetic changes likely precede mutations changes. That is, when sustained increased or decreased gene expression optimizes fitness, a gain or loss of function mutation will equivalently maximize fitness. Furthermore, genes highly expressed in normal tissue are often conserved suggesting this core set of genes is necessary for optimal function of both normal and malignant cells.

Analogous to loss of eyes in the cavefish, lung cancer cells turn off differentiated functions (e.g., oxygen transport, cilia movement, surfactant synthesis), and cell–cell interactions governing 3-dimensional tissue organization that provide no fitness benefit. While these properties contribute to whole tissue functioning, from the perspective of the evolving cancer cells, they consume needed resources while providing no direct fitness benefit. An interesting component of the “de-differentiating” phenotype is loss of genes associated with circadian entrainment. Normal somatic cells are subject to circadian coordination of physiological processes, including immune function [54]. Maintaining normal activity at night, when normal cells are quiescent, appears to provide an evolutionary benefit that outweighs the metabolic cost. This is consistent with the observation that breast cancers accelerate their growth during sleep [55].

Similarly, and as expected in rapidly proliferating and anabolic cells, NCD LUADs upregulate and conserve genes related to progression through the cell cycle, mitosis, and DNA replication. The upregulation of expression in multiple ribosome genes is also an expected property of proliferating cells, but the consistent selection of specific ribosome genes across the cohort suggests additional dynamics at the ribosome level that enhance cancer cells’ fitness or that there are important non-canonical functions for these genes that contribute to fitness.

Concordant changes in both expression and mutational selection reveals how NCD LUAD cells direct extensive niche construction, particularly through changes in the collagen composition of the ECM (increased *COL10A1, COL11A1, COL17A1, COL1A1,* and *COL7A1* with decreased *COL4A3, COL6A6, COL4A6, COL13A1,* and *CRTAC1* [Cartilage Acidic Protein]) along with a similar mix of increased and decreased function of ADAM and ADAMTS genes but uniform increase in MMP gene expression.

NCD LUAD cells evolutionarily generally down-regulate highly expressed genes related to innate immunity and antigen presentation in normal tissue with modest upregulation of some genes associated with check point inhibition. This is surprising since, on average, NCD LUAD cells contain over 300 mutations per sample indicating a potentially high immunogenicity to host immune cells. Thus, we hypothesize that the extensive ECM remodeling may generate an immune inhibitory environment through increased expression *COL1A1* [56], *COL10A1* [57], *COL11A1* [30], *COL17A1* [58], *ADAM12* [59], *ADAMTS16* [60], *ADAMDEC1* [61], and *ADAMT14* [62].

*TP53* is the most highly mutated gene in NCD LUAD yet the selection for mutations in other signaling pathways often associated with cancer populations are generally lacking [63]. Indeed, genes associated with the canonical MAPK, WNT, TGFB, PI3K, PTEN, and AKT pathways are generally down regulated and do not show evidence of evolutionary selection for mutations or conservation. However, in the absence of clear molecular drivers, we note that there is remarkably extensive evolutionary selection on ligand- and voltage-gated ion channels and ion transporters. Prior studies have shown that reductions in the negative transmembrane potential is common in cells undergoing rapid mitosis, including cancer cells, and that manipulating this potential can alter the proliferation of cancer cells [23, 64–66]. Thus, we hypothesize the net result of changes in ion channels/transport is a reduction in the transmembrane potential that sustains proliferation of NCD LUAD. That is, rather than a specific driver mutation, NK LUAD possesses a “driver phenotype.”

While the main purpose of this investigation was better understanding of the evolutionary dynamics that generate lung cancer without driver genes, we note the results have potential clinical application. Prior theoretical investigations have suggested targeting conserved genes may be equally or more effective than the traditional approach which targets gene mutations [53]. Furthermore, changes in expression, as noted above, can provide potential information on the relative fitness benefit of mutated or conserved genes. For example, a conserved gene that has significantly increased expression is probably critical for cancer cell fitness and an ideal clinical target. Similarly, genes that are frequently mutated with corresponding significant changes in expression (up or down) are also likely to be highly valuable targets for therapy.

In general, we find NCD LUADs exhibit convergence on a common malignant phenotype through a mixture of genetic, epigenetic, and ecological mechanisms of inheritance. By integrating expression and mutational data, we see clear dynamical connections among these mechanisms of inheritance within the evolutionary arc of each cancer. Thus, for example, the strong association between expression changes and selected gene mutations suggests gain and loss of function mutations often represent a “hard wiring” of genes that have already been epigenetically selected for consistent increased or decreased expression. Furthermore, widespread conservation of genes associated with transcription control, ribosomes, homeobox, and RNA Polymerase II demonstrates the critical role of translational control in evolution of the malignant phenotype in NCD LUADs. These observations suggest NCD LUADs evolve through an interconnected network of genetic, epigenetic, and ecological pathways.

### Supplementary Information

Below is the link to the electronic supplementary material.

Supplementary file1 (DOCX 225 kb)

## Data Availability

All data used in this analysis are included in the Supplemental Material.
