# Supplementary material for: Lung adenocarcinomas without driver genes converge to common adaptive strategies through diverse genetic, epigenetic, and niche construction evolutionary pathways
Source: Med Oncol. 2024 May 5;41(6):135. doi: 10.1007/s12032-024-02344-2 (PMC11070398; doi:10.1007/s12032-024-02344-2)
Supplement: Supplementary file 1 — Supplementary file1 (DOCX 225 kb) [file 12032_2024_2344_MOESM1_ESM.docx]

**Supplemental material**

**Most expressed genes in normal lung and NKD LUAD**

As a metric of the most important genes in optimizing cell cancer cell fitness, we compared f the 100 most expressed genes in normal lung and NKD LUAD. Fifty-nine (59) genes were in the 100 most expressed genes in both normal lung cells and NKD LUAD (**Supplemental Figure 1**). Genes in common are highly enriched for translation, particularly ribosomes, (P=1.3E-13), antigen processing and presentation (P=7.6E-10), protein stabilization (P=5.1E-4), platelet alpha granules (P=9.0E-4), platelet aggregation (P=2.3E-7), lysosome function (P=2.5E-5), ER to Golgi transport (P=5.6E-7), melanosome (P=1.6E-8), chaperone binding (P=2.2E-5), and negative regulation of endopeptidase (P=7.7E-5).

Of the 41 genes only in NKD LUAD, 15 encoded ribosome proteins (P=6.5E-25). Other enriched functions included ubiquitin ligase inhibitors (P=7.3E-7), protein folding in endoplasmic reticulum (P=1.3E-11), melanosome (P=1.6E-6), cadherin binding (P=5.0E-5), glycolysis (P=3.6E-3), and extracellular matrix (P=2.5E-3).

Of the 41 genes with decreased expression in NKD LUAD compared to normal, 20 are associated with the extracellular environment (P=2.5E-10), including surfactant (P=5.0E-5) complement and coagulation cascades (P4.3E-3), protease inhibitors (P=2.0E-3), antigen processing and presentation (P4.0E-4), and endoplasmic reticulum (P=1.4E-3).

Lung cancers continue to use the most expressed ribosomes in normal lung cells but increased expression of 15 additional ribosome proteins (*RPL10, RPL19, RPLP0, RPL13A, RPL30, RPL37A, RPS16, RPS9, RPL37, RPS20, RPS19, RPL11, RPL5, RPS8, RPL13*). While these may simply represent a generalized increase in proteins synthesis, the specific distribution suggests selection for specific functions. For example, *RPL5* and *RPL11* are known to stabilize P53 (1, 2) and RPL11 may inhibit c-MYC activity (3) and *RPL5* regulates colon cancer proliferation through the MAPK/ERK signaling pathway. (4). *RPL10* may be required for meiotic division in spermatogenesis (5).

Other genes among the most expressed in lung cancers cell are associated with glycolysis (P=1.5E-3), estrogen signaling (P=2.9E-3), platelet aggregation (P=3.2E-6), angiogenesis (P=1.8E-3), glycolysis (P=6.0E-5), and innate immune defense (1.3E-2). Surprisingly, 14 of the most expressed genes are associated with the melanosome (P=2.2E-15).

**Figures**


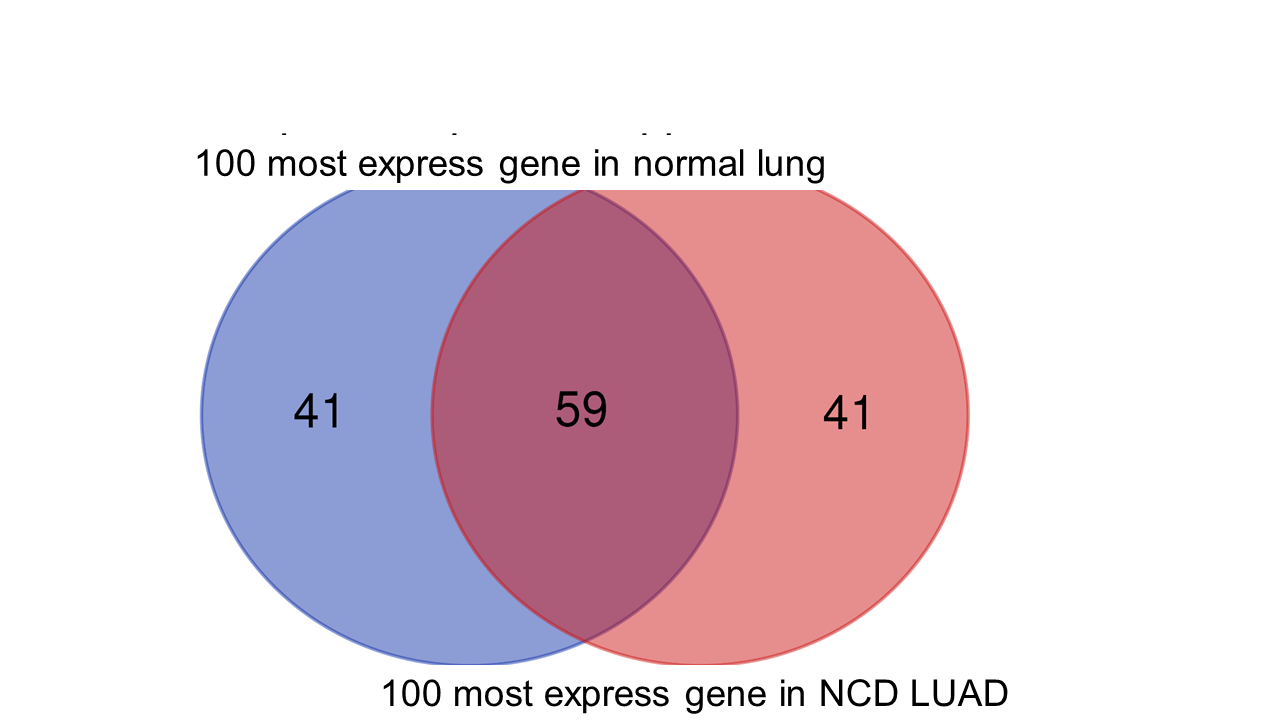


| **Names** | **total** | **elements** |
| --- | --- | --- |
| 100 most expressed genes in NKD LUAD 100 most expressed genes in normal lung | 59 | RPS11 ACTB ANXA2 SPARC ENO1 EIF4G2 RPS18 RPLP1 TMSB10 TPT1 HLA-C SFTPA2 FTH1 MYH9 EEF2 CTSB GAPDH GLUL CD74 CTSD HLA-DRA UBB CD63 S100A6 RPS4X RACK1 RPS3 RPL3 HLA-B TMBIM6 VIM APLP2 ACTG1 PSAP HSP90AB1 HLA-A RPS6 FLNA SLC34A2 A2M SFTPB PABPC1 HSPA8 GRN MYL6 RPL8 B2M GNAS TAGLN2 APP EEF1A1 FN1 RPL4 UBC HSP90AA1 ATP1A1 FTL RPL15 C3 |
| 100 most expressed genes in normal lung | 41 | ABCA3 CTSH NAPSA DHCR24 DCN RNASE1 SERPING1 TNS1 EPAS1 SFTPC CAVIN1 HLA-DPA1 HLA-DRB1 SPTBN1 CAV1 MSN CD59 PIGR LYZ CLDN18 TGM2 SCD LPCAT1 ITM2B TIMP3 CD81 RHOA MCL1 TXNIP SFTPD SERPINA1 DUSP1 SLPI AGER AHNAK SFTPA1 VWF NPC2 HLA-E EMP2 AQP1 |
| 100 most expressed genes in NKD LUAD | 41 | RPL10 HSPA5 TUBB CFL1 COL3A1 PTMA MUC1 PKM RPL19 RPLP0 CEACAM6 RPL13A RPL30 CALR LGALS3BP RPL37A ARF1 KRT8 RPS16 ALDOA CANX LDHA RPS9 HSP90B1 COL1A2 RPL37 RPS20 HNRNPA2B1 KRT18 COL1A1 PDIA3 RPL11 RPS19 EEF1G RPL5 RPS8 TUBA1B HDLBP P4HB RPL13 YWHAZ |

**Supplemental Figure 1**. Overlap of 100 most expressed genes in Normal lung and NKD LUAD. Of the 100 most highly expressed genes in normal lung cells, 59 remain highly among the most highly expressed in NK LUAD cells. Highly expressed genes unique to lung cancers include 15 encoding ribosomes proteins, genes associated with the extracellular matrix, and glycolysis. Genes associated with surfactant as well as innate immunity and antigen presentation are down regulated in lung cancer cells.

| **Genes related to transmembrane ion gradient** | | | |
| --- | --- | --- | --- |
| **Sodium Transporters** | | | |
| Gene Name | Mutated Samples  (n=313) | Distance from neutral line (Standard Deviation) | Expression change compared to normal lung  (base 2) |
| ATPase Na^+^/K^+^ transporting subunit alpha 2 (ATP1A2) | 15 | 1.1 | -3.1 |
| CF Transmembrane Conductance Regulator (CFTR) | 7 | -0.8 | -2.7 |
| FXYD domain containing ion transport regulator 1 (FXYD1) | 1 | 0.3 | -3.6 |
| sodium channel epithelial 1 subunit gamma (SCNN1G) | 6 | 0.2 | -2.3 |
| sodium voltage-gated channel alpha subunit 1 (SCN1A) | 25 | 1.0 | -3.3 |
| sodium voltage-gated channel 2 beta (SCN2B) | 2 | 0.2 | -2.5 |
| sodium voltage-gated channel alpha subunit 7 (SCN7A) | 21 | 1.8 | -3.4 |
| sodium voltage-gated channel alpha subunit 8 (SCN8A) | 17 | 0.2 | 2.1 |
| sodium voltage-gated channel beta subunit 4 (SCN4B) | 1 | -0.3 | -3.3 |
| Sodium Channel Epithelial 1 Subunit Beta (SCNN1B) | 3 | -0.4 | -1.8 |
| Sodium Channel Epithelial 1 Subunit Gamma (SCNN1G) | 6 | 0.2 | -2.3 |
| solute carrier family 13 member 2 (SLC13A2) | 3 | -0.4 | -2.3 |
| solute carrier family 5 member 1 (SLC5A1) | 9 | 0.8 | -2.7 |
| solute carrier family 5 member 4 (SLC5A4) | 8 | 0.6 | -2.1 |
| solute carrier family 5 member 7 (SLC5A7) | 8 | 0.8 | -2.6 |
| solute carrier family 5 member 8 (SLC5A8) | 12 | 1.4 | -2.4 |
| solute carrier family 5 member 9 (SLC5A9) | 9 | 0.7 | -3.6 |
| sodium leak channel, non-selective (NALCN) | 49 | 3.3 | -0.6 |
| **Glutamate Receptors** | | | |
| glutamate ionotropic receptor AMPA type subunit 1 (GRIA1) | 11 | 0.7 | -5.2 |
| glutamate ionotropic receptor AMPA type subunit 4(GRIA4) | 22 | 2.1 | 0.3 |
| glutamate ionotropic receptor delta type subunit 1 (GRID1) | 22 | 2.0 | -1.9 |
| glutamate ionotropic receptor kainate type subunit 2 (GRIK2) | 26 | 2.5 | 1.4 |
| glutamate ionotropic receptor kainate type subunit 4 (GRIK4) | 11 | 0.9 | -3.0 |
| glutamate ionotropic receptor NMDA 1 GRIN1 | 5 | -0.2 | 2.4 |
| glutamate ionotropic receptor NMDA type subunit 2A (GRIN2A) | 32 | 2.3 | -0.7 |
| glutamate ionotropic receptor NMDA type subunit 3A (GRIN3A) | 27 | 2.6 | -0.3 |
| **K^+^ and Ca^++^ voltage gated ion channels** | | | |
| calcium voltage-gated channel subunit alpha1 B (CACNA1B) | 22 | 1.3 | 1.0 |
| calcium voltage-gated channel subunit alpha1 C (CACNA1C) | 33 | 2.1 | -1.1 |
| calcium voltage-gated channel subunit alpha1 D (CACNA1D) | 12 | -0.8 | -1.4 |
| calcium voltage-gated channel subunit alpha1 E (CACNA1E) | 51 | 3.2 | 1.6 |
| Calcium Voltage-Gated Channel Subunit Alpha1 S (CACNA1S) | 15 | 0.0 | -2.6 |
| Calcium Voltage-Gated Channel Auxiliary Subunit Alpha2delta 2 (CACNA2D2) | 0 | -3.1 | -4.3 |
| Calcium Voltage-Gated Channel Auxiliary Subunit Beta 4 (CACNB4) | 5 | 0.4 | -2.4 |
| Calcium Voltage-Gated Channel Auxiliary Subunit Gamma 4 (CACNG4) | 5 | 0.8 | -2.2 |
| Calcium Voltage-Gated Channel Auxiliary Subunit Gamma 6 (CACNG6) | 5 | 1.5 | -2.4 |
| hyperpolarization activated cyclic nucleotide gated potassium channel 1 (HCN1) | 39 | 4.0 | 0.3 |
| hyperpolarization activated cyclic nucleotide gated potassium channel 2 (HCN2) | 1 | -1.4 | 1.6 |
| hyperpolarization activated cyclic nucleotide gated potassium channel 3 (HCN3) | 4 | -0.3 | 1.5 |
| Potassium Voltage-Gated Channel Subfamily A Member 10 (KCNA10) | 8 | 0.9 | -1.2 |
| Potassium Voltage-Gated Channel Subfamily A Member 3 (KCNA3) | 7 | 0.8 | -1.6 |
| Potassium Voltage-Gated Channel Subfamily A Member 4 (KCNA4) | 20 | 2.3 | -4.3 |
| Potassium Voltage-Gated Channel Subfamily A Member 5 (KCNA5) | 17 | 2.4 | -2.3 |
| Potassium Voltage-Gated Channel Subfamily A Member 6 (KCNA6) | 6 | 0.5 | -1.0 |
| Potassium Voltage-Gated Channel Subfamily A Regulatory Beta Subunit 1 (KCNAB1) | 8 | 1.1 | -2.0 |
| Potassium Voltage-Gated Channel Subfamily A Regulatory Beta Subunit 2 (KCNAB2) | 4 | 0.9 | -1.2 |
| Potassium Voltage-Gated Channel Subfamily B Member 1 (KCNB1) | 19 | 2.0 | -1.4 |
| Potassium Voltage-Gated Channel Subfamily D Member 2 (KCND2) | 16 | 1.9 | 1.6 |
| Potassium Voltage-Gated Channel Subfamily D Member 3 (KCND3) | 11 | 1.2 | -1.1 |
| Potassium Voltage-Gated Channel Subfamily E Member 1 (KCNE1) | 1 | 0.1 | -3.0 |
| Potassium Voltage-Gated Channel Subfamily E Member 4 (KCNE4) | 1 | -0.1 | 1.4 |
| Potassium Voltage-Gated Channel Subfamily G Member 2 (KCNG2) | 1 | -0.5 | 1.4 |
| Potassium Voltage-Gated Channel Subfamily H Member 3 (KCNH3) | 9 | 0.2 | -1.2 |
| Potassium Voltage-Gated Channel Subfamily H Member 6 (KCNH6) | 12 | 0.7 | -1.4 |
| Potassium Voltage-Gated Channel Interacting Protein 1 (KCNIP1) | 3 | 0.6 | -2.1 |
| Potassium Inwardly Rectifying Channel Subfamily J Member 10 (KCNJ10) | 5 | 0.6 | 1.9 |
| Potassium Inwardly Rectifying Channel Subfamily J Member 12 (KCNJ12) | 21 | 2.9 | -1.3 |
| (Potassium Inwardly Rectifying Channel Subfamily J Member 15 (KCNJ15) | 3 | 0.1 | -2.6 |
| Potassium Inwardly Rectifying Channel Subfamily J Member 16 (KCNJ16) | 7 | 0.9 | -2.2 |
| Potassium Inwardly Rectifying Channel Subfamily J Member 2 (KCNJ2) | 9 | 1.3 | -1.1 |
| Potassium Inwardly Rectifying Channel Subfamily J Member 3 (KCNJ3) | 23 | 3.0 | -0.6 |
| Potassium Inwardly Rectifying Channel Subfamily J Member 5 (KCNJ5) | 3 | 0.0 | -2.4 |
| Potassium Inwardly Rectifying Channel Subfamily J Member 8 (KCNJ8) | 6 | 0.7 | -1.7 |
| Potassium Two Pore Domain Channel Subfamily K Member 1 (KCNK1) | 3 | 0.3 | 0.9 |
| Potassium Two Pore Domain Channel Subfamily K Member 10 (KCNK10) | 16 | 2.0 | 0.4 |
| Potassium Two Pore Domain Channel Subfamily K Member 13 (KCNK13) | 10 | 2.1 | 0.4 |
| Potassium Two Pore Domain Channel Subfamily K Member 17 (KCNK17) | 4 | 0.8 | -2.0 |
| Potassium Two Pore Domain Channel Subfamily K Member 2 (KCNK2) | 16 | 2.3 | 0.7 |
| Potassium Two Pore Domain Channel Subfamily K Member 3 (KCNK3) | 2 | 0.1 | -3.1 |
| Potassium Two Pore Domain Channel Subfamily K Member 9 (KCNK9) | 11 | 1.8 | 1.2 |
| Potassium Calcium-Activated Channel Subfamily M Regulatory Beta Subunit 2 (KCNMB2) | 6 | 1.2 | -2.3 |
| Potassium Calcium-Activated Channel Subfamily M Regulatory Beta Subunit 3 (KCNMB3) | 2 | 0.0 | 1.2 |
| Potassium Calcium-Activated Channel Subfamily N Member 4 (KCNN4) | 5 | 0.6 | 2.7 |
| Potassium Voltage-Gated Channel Subfamily Q Member 1 (KCNQ1) | 2 | -0.7 | -1.2 |
| Potassium Voltage-Gated Channel Subfamily Q Member 2 (KCNQ2) | 14 | 1.9 | 1.3 |
| Potassium Voltage-Gated Channel Subfamily Q Member 3 (KCNQ3) | 2 | -1.2 | 1.9 |
| Potassium Voltage-Gated Channel Subfamily Q Member 5 (KCNQ5) | 7 | 0.0 | 1.5 |
| Potassium Channel Regulator (KCNRG) | 1 | -0.4 | -1.9 |
| Potassium Voltage-Gated Channel Modifier Subfamily S Member 1 (KCNS1) | 0 | -2.0 | -2.0 |
| Potassium Sodium-Activated Channel Subfamily T Member 2 (KCNT2) | 41 | 3.6 | -3.4 |
| **Gamma-aminobutyric Acid Receptors** | | | |
| gamma-aminobutyric acid type A receptor subunit alpha2 (GABRA2) | 21 | 2.8 | -0.5 |
| gamma-aminobutyric acid type A receptor subunit alpha3 (GABRA3) | 11 | 1.4 | 2.2 |
| gamma-aminobutyric acid type A receptor subunit beta2 (GABRB2) | 4 | 0.1 | -1.6 |
| gamma-aminobutyric acid type A receptor subunit beta3 (GABRB3) | 23 | 3.2 | -1.0 |
| **Cholinergic receptors** | | | |
| Cholinergic Receptor Muscarinic 1 (CHRM1) | 2 | -0.5 | -4.7 |
| Cholinergic Receptor Muscarinic 2 (CHRM2)) | 25 | 3.2 | -2.8 |
| Cholinergic Receptor Muscarinic 3 (CHRM3) | 17 | 2.1 | -1.4 |
| **Synaptic organization** | | | |
| Neuregulin 1 (NRG1) | 17 | 2.1 | -2.2 |
| Neuregulin 2 (NRG2) | 4 | 0.2 | -1.2 |
| Neuregulin 3 (NRG3) | 19 | 2.4 | -3.0 |
| Neuroligin 1 (NLGN1) | 20 | 2.0 | -1.2 |
| Neuroligin 4 X-Linked (NLGN4X) | 30 | 3.1 | -1.3 |
| **Serotonin Transporters (Na^+^ and Cl- dependent)** | | | |
| Solute Carrier Family 6 Member 4 (SLC6A4) | 4 | -0.2 | -7.9 |
| Solute Carrier Family 6 Member 13 (SLC6A13) | 5 | 0.2 | -3.0 |
| Solute Carrier Family 6 Member 20 (SLC6A20) | 2 | -0.7 | -2.3 |
| Solute Carrier Family 6 Member 16 (SLC6A16) | 8 | 0.5 | -1.4 |
| **Other ion channels** | | | |
| ryanodine receptor 2 (RYR2) | 125 | 5.6 | -1.8 |
| ryanodine receptor 3 (RYR3) | 66 | 2.2 | -0.9 |
| transient receptor potential cation channel subfamily A member 1 (TRPA1) | 36 | 3.1 | 1.3 |
| transient receptor potential cation channel subfamily C member 3 (TRPC3) | 10 | 0.6 | -1.6 |
| Transient Receptor Potential Cation Channel Subfamily C Member 6 (TRPC6) | 16 | 1.5 | -1.6 |
| Transient Receptor Potential Cation Channel Subfamily M Member 1 (TRPM1) | 21 | 1.0 | -1.0 |
| Transient Receptor Potential Cation Channel Subfamily M Member 2 (TRPM2) | 14 | 0.5 | 1.5 |
| Transient Receptor Potential Cation Channel Subfamily M Member 8 (TRPM8) | 13 | 0.7 | 3.5 |
| Transient Receptor Potential Cation Channel Subfamily V Member 1 (TRPV1) | 4 | -0.1 | 1.2 |
| Transient Receptor Potential Cation Channel Subfamily V Member 2 (TRPV2) | 9 | 0.7 | -2.0 |
| Transient Receptor Potential Cation Channel Subfamily V Member 3 (TRPV3) | 6 | 0.1 | 1.2 |
| chloride voltage-gated channel 1 (CLCN1) | 28 | 2.8 | 0.5 |
| chloride voltage-gated channel 2 (CLCN2) | 2 | -1.3 | 1.5 |
| chloride voltage-gated channel 4 (CLCN4) | 8 | 0.5 | -1.2 |

**Supplemental Table 1.**  Genes related to Membrane ion channels and ion transporters.

| **Genes related to Niche Construction** | | | | | | |
| --- | --- | --- | --- | --- | --- | --- |
| Gene name | | Number of samples mutated | Standard deviation from neutral line | | Expression change (base 2) | |
| Extracellular matrix remodeling | | | | | | |
| ADAM metallopeptidase with thrombospondin type 1 motif 1(ADAMTS1) | | 13 | 1.2 | | -2.4 | |
| ADAM metallopeptidase with thrombospondin type 1 motif 2(ADAMTS2) | | 24 | 2.4 | | -0.1 | |
| ADAM metallopeptidase with thrombospondin type 1 motif 12 (ADAMTS12) | | 62 | 4.4 | | 1.2 | |
| ADAM metallopeptidase with thrombospondin type 1 motif 14(ADAMTS14) | | 16 | 1.1 | | 2.5 | |
| ADAM metallopeptidase with thrombospondin type 1 motif 15(ADAMTS15) | | 12 | 0.8 | | -2.2 | |
| ADAM metallopeptidase with thrombospondin type 1 motif 16(ADAMTS16) | | 37 | 3.2 | | 2.7 | |
| ADAM metallopeptidase with thrombospondin type 1 motif 17 (ADAMTS17) | | 15 | 1.5 | | -1.0 | |
| ADAM metallopeptidase with thrombospondin type 1 motif 18 (ADAMTS18) | | 21 | 1.6 | | 1.5 | |
| ADAM metallopeptidase with thrombospondin type 1 motif 19 (ADAMTS19) | | 21 | 1.9 | | -0.2 | |
| ADAM metallopeptidase with thrombospondin type 1 motif 16 (ADAMTS16) | | 24 | 2.4 | | -0.1 | |
| ADAM metallopeptidase with thrombospondin type 1 motif 20 (ADAMTS20) | | 44 | 3.3 | | 1.1 | |
| ADAM metallopeptidase with thrombospondin type 1 motif5 (ADAMTS5) | | 23 | 2.5 | | 0.0 | |
| ADAM metallopeptidase with thrombospondin type 1 motif 8 (ADAMTS8) | | 5 | 0.1 | | -4.7 | |
| ADAMTS like 3 (ADAMTSL3) | | 27 | 1.5 | | -3.0 | |
| ADAMTS like 4 (ADAMTSL4) | | 7 | -0.3 | | -2.3 | |
| Bone morphogenetic protein 2 (BMP2) | | 1 | -0.7 | | -2.4 | |
| Bone morphogenetic protein 5 (BMP5) | | 3 | -0.1 | | -1.8 | |
| Bone morphogenetic protein 6 (BMP6) | | 8 | 1.3 | | -1.6 | |
| Bone morphogenetic protein 8a (BMP8A) | | 1 | -0.6 | | 2.5 | |
| Bone morphogenetic protein 8b (BMP8B) | | 0 | -1.7 | | 1.0 | |
| BMP Binding Endothelial Regulator (BMPER) | | 17 | 1.9 | | -3.5 | |
| MAM domain containing 2 (MAMDC2) | | 3 | -0.5 | | -3.7 | |
| SPARC (osteonectin), cwcv and kazal like domains proteoglycan 1 (SPOCK1) | | 9 | 1.4 | | 1.9 | |
| SPARC (osteonectin), cwcv and kazal like domains proteoglycan 2 (SPOCK2) | | 2 | -0.1 | | -3.4 | |
| SPARC like 1 (SPARCL1) | | 5 | 0.0 | | -2.1 | |
| TIMP metallopeptidase inhibitor 3(TIMP3) | | 1 | -2.2 | | -2.2 | |
| Wnt family member 3A (WNT3A) | | 6 | 1.1 | | -5.0 | |
| Wnt family member 3 (WNT3) | | 0 | -1.6 | | 1.7 | |
| Wnt family member 7A (WNT7A) | | 11 | 1.8 | | -4.1 | |
| Wnt family member 11 (WNT11 | | 3 | 0.6 | | -1.7 | |
| cartilage acidic protein 1(CRTAC1) | | 2 | -0.8 | | -4.3 | |
| cartilage intermediate layer protein 2 (CILP2) | | 13 | 1.0 | | 3.5 | |
| cartilage oligomeric matrix protein (COMP) | | 3 | -0.4 | | 2.9 | |
| Cochlin (COCH) | | 2 | -0.7 | | 2.9 | |
| collagen type I alpha 1 chain (COL1A1) | | 16 | 0.8 | | 2.8 | |
| collagen type III alpha 1 chain (COL3A1) | | 43 | 3.6 | | 2.4 | |
| collagen type IV alpha 3 chain (COL4A3) | | 8 | -0.7 | | -2.7 | |
| collagen type IV alpha 4 chain (COL4A4) | | 18 | 0.6 | | -2.0 | |
| collagen type IV alpha 6 chain (COL4A6) | | 15 | 0.2 | | -2.2 | |
| collagen type VI alpha 3 chain (COL6A3) | | 41 | 1.3 | | 1.0 | |
| collagen type VI alpha 6 chain (COL6A6) | | 35 | 1.9 | | -3.9 | |
| collagen type VII alpha 1 chain (COL7A1) | | 21 | -0.2 | | 3.0 | |
| collagen type X alpha 1 chain (COL10A1) | | 6 | 0.2 | | 4.5 | |
| collagen type XI alpha 1 chain (COL11A1) | | 82 | 5.4 | | 6.0 | |
| collagen type XVII alpha 1 chain (COL17A1) | | 7 | -0.8 | | 3.0 | |
| collagen type XXII alpha 1 chain (COL22A1) | | 44 | 2.6 | | 2.6 | |
| Collagen Triple Helix Repeat Containing 1 (CTHRC1) | | 0 | -1.3 | | 3.9 | |
| Epiphycan (EPYC) | | 5 | 0.8 | | 2.3 | |
| fibrillin 2 (FBN2) | | 57 | 2.8 | | 0.5 | |
| fibrillin 3 (FBN3) | | 21 | 0.1 | | -2.8 | |
| fibulin 5 (FBLN5) | | 4 | 0.3 | | -2.1 | |
| Latent Transforming Growth Factor Beta Binding Protein 1 (LTBP1) | | 38 | 2.8 | | -0.1 | |
| Latent Transforming Growth Factor Beta Binding Protein 2 (LTBP2) | | 18 | 0.7 | | -1.0 | |
| Gliomedin (GLDN) | | 4 | 0.2 | | -3.1 | |
| heparanase 2 (inactive) (HPSE2) | | 6 | 0.5 | | -2.7 | |
| laminin subunit gamma 3 (LAMC3) | | 12 | 0.2 | | -2.1 | |
| lipoprotein lipase (LPL) | | 4 | 0.2 | | -3.4 | |
| Membrane Metalloendopeptidase (MME) | | 12 | 1.1 | | -3.5 | |
| matrix metallopeptidase 1 (MMP1) | | 6 | 0.6 | | 4.7 | |
| matrix metallopeptidase 10 (MMP10) | | 6 | 0.6 | | 2.2 | |
| matrix metallopeptidase 11 (MMP11) | | 2 | -0.4 | | 5.2 | |
| matrix metallopeptidase 13 (MMP13) | | 7 | 0.8 | | 4.6 | |
| matrix metallopeptidase 16 (MMP16) | | 28 | 3.2 | | 0.8 | |
| matrix metallopeptidase 17 (MMP17) | | 6 | 0.7 | | 2.1 | |
| matrix metallopeptidase 3 (MMP3) | | 4 | 0.2 | | 2.7 | |
| matrix metallopeptidase 9 (MMP9) | | 9 | 1.1 | | 2.5 | |
| microfibril associated protein 4 (MFAP4) | | 2 | 0.1 | | -3.2 | |
| Microfibril Associated Protein 3 Like (MFAP3L) | | 8 | 1.2 | | -2.4 | |
| multimerin 1 (MMRN1) | | 26 | 2.0 | | -4.7 | |
| multimerin 2 (MMRN2) | | 5 | -0.2 | | -2.2 | |
| Myocilin (MYOC) | | 12 | 1.6 | | -4.7 | |
| Nephronectin (NPNT) | | 8 | 0.9 | | -2.4 | |
| Osteoglycin (OGN) | | 2 | -0.1 | | -3.4 | |
| Retbindin (RTBDN) | | 2 | 0.2 | | 2.1 | |
| Serine Protease 1 (PRSS1) | | 16 | 2.8 | | 1.9 | |
| Serine Protease 12 (PRSS12) | | 2 | -1.2 | | -2.2 | |
| Serine Protease 3 (PRSS3) | | 0 | -1.5 | | 2.7 | |
| Serine Protease 35 (PRSS35) | | 4 | 0.3 | | -2.7 | |
| Serine Protease 50 (PRSS50) | | 0 | -1.7 | | 2.2 | |
| Serine Protease 53 (PRSS53) | | 2 | -0.5 | | 1.4 | |
| somatomedin B and thrombospondin type 1 domain containing (SBSPON) | | 3 | 0.6 | | -3.2 | |
| tenascin R (TNR) | | 63 | 4.8 | | -3.0 | |
| tenascin XB (TNXB) | | 18 | -1.4 | | -3.3 | |
| von Willebrand factor (VWF) | | 19 | -0.4 | | -2.5 | |
| Glucose metabolism and extracellular acidosis | | | | | | |
| hypoxia inducible factor 1 subunit alpha (HIF1A) | 0 | | | -2.6 | | 0.6 |
| Endothelial PAS Domain Protein 1 (EPAS1) (HIF2A) | 9 | | | 0.5 | | -2.7 |
| hypoxia inducible factor 3 subunit alpha (HIF3A) | 8 | | | 0.8 | | -2.3 |
| SLC2A1 (GLUT 1) | 0 | | | -2.0 | | 3.1 |
| solute carrier family 2 member 12 (SLC2A12) | 3 | | | 8.1 | | -1.1 |
| solute carrier family 2 member 14 (SLC2A14) | 6 | | | 6.0 | | -1.3 |
| solute carrier family 2 member 3 (SLC2A3) | 9 | | | 11.5 | | -1.5 |
| solute carrier family 2 member 5 (SLC2A5) (GLUT5) | 3 | | | 0.0 | | 3.0 |
| Carbonic Anhydrase 1 (CA1) | 3 | | | 0.5 | | -2.2 |
| Carbonic Anhydrase 10 (CA10) | 9 | | | 1.5 | | 0.8 |
| Carbonic Anhydrase 11 (CA11) | 0 | | | -1.6 | | 0.0 |
| Carbonic Anhydrase 2 (CA2) | 1 | | | -0.3 | | -2.5 |
| Carbonic Anhydrase 3 (CA3) | 6 | | | 1.2 | | -2.8 |
| Carbonic Anhydrase 4 (CA4) | 4 | | | 0.6 | | -5.7 |
| Carbonic Anhydrase 8 (CA8) | 5 | | | 0.9 | | -1.3 |
| Carbonic Anhydrase 9 (CA9) | 3 | | | -0.1 | | 4.9 |
| Lactate Dehydrogenase A (LDHA) | 2 | | | -0.2 | | 1.3 |
| Glyceraldehyde-3-Phosphate Dehydrogenase (GAPDH) | 1 | | | -0.6 | | 1.6 |
| Ras Related Glycolysis Inhibitor And Calcium Channel Regulator (RRAD) | 1 | | | -0.3 | | -1.7 |
| Enolase 1 (ENO1) | 5 | | | 0.5 | | 1.2 |
| Enolase 2 (ENO2) | 0 | | | -1.8 | | 0.4 |
| Enolase 3 (ENO3) | 2 | | | -0.4 | | 1.6 |
| Phosphofructokinase, Platelet (PFKP) | 4 | | | -0.4 | | 1.9 |
| Aldolase, Fructose-Bisphosphate A (ALDOA) | 4 | | | 0.4 | | 1.2 |
| Glucose-6-Phosphate Isomerase (GPI) | 8 | | | 0.9 | | 1.1 |
| Angiogenesis | | | | | | |
| Vascular Endothelial Growth Factor A (VEGFA) | 2 | | | 0.0 | | 0.1 |
| Vascular Endothelial Growth Factor B (VEGFB) | 0 | | | -1.2 | | -0.1 |
| Vascular Endothelial Growth Factor C (VEGFC) | 13 | | | 2.1 | | -0.8 |
| Vascular Endothelial Growth Factor D (VEGFD) | 1 | | | -0.6 | | -5.0 |
| Kinase Insert Domain Receptor (KDR) | 31 | | | 2.3 | | -1.2 |
| Fibroblast Growth Factor 2 (FGF2) | 2 | | | 0.5 | | -2.4 |
| TEK Receptor Tyrosine Kinase TEK | 3 | | | -1.2 | | -3.5 |
| Thrombospondin 2 (THBS2) | 22 | | | 1.9 | | 2.6 |
| Vascular Maturation | | | | | | |
| angiopoietin 1 (ANGPT1) | 7 | | | 0.7 | | -2.8 |
| angiopoietin 4 (ANGPT4) | 9 | | | 1.2 | | -4.1 |
| angiopoietin like 1 (ANGPTL1) | 1 | | | -1.0 | | -3.1 |
| angiopoietin like 5 (ANGPTL5) | 6 | | | 0.8 | | -2.4 |
| angiopoietin like 7 (ANGPTL7) | 1 | | | -0.6 | | -4.4 |

**Table 2.** Strong positive and negative selection of genes related to the extracellular matrix and angiogenesis illustrate extensive remodeling.

| Gene name | Number of samples mutated | Standard deviation from neutral line | Expression change (base 2) |
| --- | --- | --- | --- |
| **Innate immunity and antigen presentation** | | | |
| leukocyte immunoglobulin like receptor A1 (LILRA1) | 16 | 2.2 | -1.7 |
| leukocyte immunoglobulin like receptor A2 (LILRA2) | 9 | 1.2 | -2.1 |
| leukocyte immunoglobulin like receptor A3 (LILRA3) | 9 | 1.3 | -1.1 |
| leukocyte immunoglobulin like receptor A5 (LILRA5) | 4 | 0.6 | -2.3 |
| leukocyte immunoglobulin like receptor A6 (LILRA6) | 9 | 1.2 | -1.7 |
| leukocyte immunoglobulin like receptor B1 (LILRB1) | 18 | 2.2 | -0.9 |
| leukocyte immunoglobulin like receptor B2 (LILRB2) | 17 | 2.2 | -0.8 |
| leukocyte immunoglobulin like receptor B3 (LILRB3) | 1 | -1.2 | -1.5 |
| Interleukin 11 Receptor Subunit Alpha (IL11RA) * | 1 | -0.8 | -1.2 |
| Interleukin 12 Receptor Subunit Beta 2 (IL12RB2) * | 8 | 0.3 | 1.1 |
| Interleukin 16 (IL16) * | 18 | 1.0 | -1.1 |
| Interleukin 17C (IL17C) * | 0 | -1.2 | 1.6 |
| Interleukin 17D (IL17D*_ | 1 | 0.2 | -2.0 |
| Interleukin 17 Receptor D (IL17RD) * | 5 | 0.1 | 1.1 |
| Interleukin 17 Receptor E (IL17RE) * | 2 | -0.7 | -1.0 |
| Interleukin 17 Receptor E Like (IL17REL) * | 1 | -0.4 | 1.6 |
| Interleukin 18 Receptor 1 (IL18R1) * | 7 | 0.6 | -1.5 |
| Interleukin 18 Receptor Accessory Protein (IL18RAP) * | 15 | 1.8 | -1.0 |
| Interleukin 1A (IL1A) * | 3 | 0.4 | -2.5 |
| Interleukin 1B (IL1B) * | 2 | 0.0 | -1.4 |
| Interleukin 1 Receptor Type 1 (IL1R1) | 0 | -2.1 | -1.0 |
| Interleukin 1 Receptor Like 1 (IL1RL1) * | 8 | 0.8 | -3.8 |
| Interleukin 1 Receptor Like 2 (IL1RL2) * | 2 | -0.7 | 2.5 |
| Interleukin 20 Receptor Subunit Alpha 2 (IL20RA) * | 2 | -0.6 | -2.2 |
| Interleukin 20 Receptor Subunit Beta 2 (IL20RB) * | 4 | 0.6 | 2.3 |
| Interleukin 22 Receptor Subunit Alpha 2 (IL22RA2) * | 2 | 0.0 | 2.8 |
| Interleukin 23 Subunit Alpha (IL23A) * | 0 | -1.1 | 2.0 |
| Interleukin 2 Receptor Subunit Alpha (IL2RA) * | 6 | 1.1 | 1.5 |
| Interleukin 31 Receptor Subunit Alpha (IL31RA) * | 6 | 0.0 | 2.6 |
| Interleukin 33 (IL33) * | 1 | -0.4 | -2.8 |
| Interleukin 34 (IL34) * | 0 | -1.3 | -1.5 |
| Interleukin 36 Gamma (IL36G) * | 5 | 1.2 | 1.5 |
| Interleukin 36 Receptor Antagonist (IL36RN) * | 3 | 0.8 | 3.0 |
| Interleukin 37 (IL37) * | 4 | 0.8 | 2.2 |
| Interleukin 3 Receptor Subunit Alpha (IL3RA) * | 6 | 0.8 | -1.8 |
| Interleukin 4 Induced 1 (IL4I1) * | 6 | 0.8 | 1.9 |
| Interleukin 5 Receptor Subunit Alpha (IL5RA) * | 8 | 1.1 | -2.3 |
| Interleukin 6 (IL6) * | 1 | -0.2 | -2.3 |
| Interleukin 6 Receptor (IL6R) * | 4 | 0.2 | -1.2 |
| Interleukin 6 Cytokine Family Signal Transducer (IL6ST) * | 1 | -1.8 | -1.2 |
| Interleukin 17 Receptor A (IL7RA) * | 13 | 1.8 | -2.4 |
| Butyrophilin Like 3 (BTNL3) * | 4 | 0.2 | -1.2 |
| Butyrophilin Like 8 (BTNL8) * | 8 | 1.0 | -2.8 |
| Butyrophilin Like 9 (BTNL9) | 3 | 0.0 | -4.1 |
| Cathepsin G (CTSG)* | 6 | 1.2 | -2.1 |
| Cathepsin H (CTSH)* | 0 | -1.6 | -1.6 |
| Cathepsin V (CTSV)* | 1 | -0.6 | 2.2 |
| Cathepsin G (CTSG)* | 6 | 1.2 | -2.1 |
| Cathepsin H (CTSH)* | 0 | -1.6 | -1.6 |
| Cathepsin W (CTSW)* | 0 | -1.7 | -1.2 |
| macrophage receptor with collagenous structure (MARCO) | 15 | 2.1 | -4.1 |
| Caveolin 1 (CAV1) * | 0 | -1.1 | -3.9 |
| Caveolin 2 (CAV2) * | 0 | -1.0 | -2.7 |
| Caveolin 3 (CAV3) * | 3 | 0.8 | -3.1 |
| Caveolae Associated Protein 1 (CAVIN1) * | 1 | -0.6 | -2.2 |
| complement C6 (C6) | 19 | 1.7 | -3.1 |
| complement C7 (C7) | 24 | 2.7 | -2.9 |
| complement C8 beta chain (C8B) | 10 | 1.1 | -4.5 |
| complement factor properdin (CFP) | 4 | 0.7 | -2.4 |
| Collectin Subfamily Member 10 (COLEC10) * | 3 | 0.4 | -3.0 |
| Collectin Subfamily Member 12 (COLEC12) * | 7 | 0.3 | -2.3 |
| Endoplasmic Reticulum Aminopeptidase 1 (ERAP1) | 0 | -2.8 | -0.4 |
| Major Histocompatibility Complex, Class I, A (HLA-A) | 4 | 0.4 | 0.0 |
| Major Histocompatibility Complex, Class I, B (HLA-B) | 2 | -0.2 | -0.4 |
| Major Histocompatibility Complex, Class I, C (HLA-C) | 3 | 0.2 | -0.5 |
| Major Histocompatibility Complex, Class II, DMAlpha (HLA-DMA) | 0 | -1.4 | -0.9 |
| Major Histocompatibility Complex, Class II, DMBeta (HLA-DMB) | 0 | -1.4 | -0.8 |
| Major Histocompatibility Complex, Class II, DOAlpha (HLA-DOA) | 3 | 0.5 | -1.4 |
| Major Histocompatibility Complex, Class II, DOBeta (HLA-DOB) | 0 | -1.4 | 0.1 |
| Major Histocompatibility Complex, Class II, DPAlpha1 (HLA-DPA1) | 0 | -1.4 | -1.3 |
| Major Histocompatibility Complex, Class II, DPBeta1 (HLA-DPB1) | 0 | -1.4 | -1.2 |
| Major Histocompatibility Complex, Class II, DQAlpha1 (HLA-DQ1) | 0 | -1.3 | -1.2 |
| Major Histocompatibility Complex, Class II, DQA2 (HLA-DQAlpha2) | 3 | 0.5 | -1.1 |
| Major Histocompatibility Complex, Class II, DQB1 (HLA-DQBeta1) | 5 | 1.0 | -1.1 |
| Major Histocompatibility Complex, Class II, DQB2 (HLA-DQB2) | 3 | 1.1 | -0.6 |
| Major Histocompatibility Complex, Class II, DRAlpha (HLA-DRA) | 2 | 0.1 | -1.3 |
| Major Histocompatibility Complex, Class II, DRB1 (HLA-DRBeta1) | 0 | -1.4 | -1.3 |
| Major Histocompatibility Complex, Class II, DRBeta5 (HLA-DRB5) | 0 | -1.4 | -1.6 |
| Major Histocompatibility Complex, Class I, E (HLA-E) | 0 | -1.6 | -1.2 |
| Major Histocompatibility Complex, Class I, F (HLA-F) | 0 | -1.8 | -0.4 |
| Major Histocompatibility Complex, Class I, G (HLA-G) | 2 | -0.2 | -0.6 |
| **Inflammation** | | | |
| NLR Family Pyrin Domain Containing 1 (NLRP1) | 11 | 0.0 | -0.7 |
| NLR Family Pyrin Domain Containing11 (NLRP11) | 12 | 0.6 | 1.0 |
| NLR Family Pyrin Domain Containing 12 (NLRP12) | 22 | 1.8 | -1.4 |
| NLR Family Pyrin Domain Containing14 (NLRP14) | 28 | 2.4 | -0.8 |
| NLR Family Pyrin Domain Containing 2 (NLRP2) | 15 | 1.0 | -0.3 |
| NLR Family Pyrin Domain Containing 3 (NLRP3) | 35 | 3.2 | -0.9 |
| NLR Family Pyrin Domain Containing 4 (NLRP4) | 21 | 1.8 | 0.4 |
| NLR Family Pyrin Domain Containing 6 (NLRP6) | 8 | 0.9 | 0.1 |
| NLR Family Pyrin Domain Containing 7 NLRP7 | 22 | 1.9 | 0.8 |
| NLR Family Pyrin Domain Containing 9 (NLRP9) | 10 | 0.4 | 0.0 |
| Serine/Threonine Kinase 11 (STK11) | 27 | 5.5 | -0.3 |
| phospholipase A2 group IB (PLA2G1B) * | 1 | 0.0 | -4.6 |
| phospholipase A2 group I2B(PLA2G12B) * | 4 | 1.0 | -1.6 |
| phospholipase A2 group G2A (PLA2G2A) * | 4 | 1.1 | -1.3 |
| phospholipase A2 group G3(PLA2G3) * | 5 | 0.4 | -3.0 |
| phospholipase A2 group G4F (PLA2G4F) * | 3 | -0.7 | -3.6 |
| phospholipase A2 group (G5PLA2G5) * | 0 | -0.9 | -1.4 |
| **Check point proteins** | | | |
| CD274 Molecule (CD274) | 3 | 0.4 | -1.2 |
| Programmed Cell Death 1 (PDCD1) * | 4 | 0.8 | 1.0 |
| Programmed Cell Death 1 (PDCD1LG2) * | 2 | 0.0 | -1.3 |
| Programmed Cell Death 2 Like (PDCD2L) * | 1 | -0.6 | 1.4 |
| Programmed Cell Death 6 (PDCD6) * | 0 | -1.1 | 1.0 |
| Cytotoxic T-Lymphocyte Associated Protein 4 (CTLA4) * | 2 | 0.2 | 1.1 |
| Lymphocyte activating 3  ( LAG3) | 2 | -0.5 | 1.1 |
| **Membrane attack complex** | | | |
| BMP/retinoic acid inducible neural specific 2 (BRINP2) | 23 | 2.4 | 0.2 |
| BMP/retinoic acid inducible neural specific 3 (BRINP3) | 39 | 3.9 | -1.1 |
| astrotactin 1 (ASTN1) | 52 | 4.2 | -1.1 |
| astrotactin 2 (ASTN2) | 36 | 3.2 | 0.0 |
| complement C7 (C7) | 24 | 2.7 | -2.9 |
| **CD proteins** | | | |
| CD74 | 0 | -1.5 | -1.0 |
| CD44 | 5 | -0.1 | -1.2 |
| CD68 | 0 | -1.6 | -1.6 |
| CD55 | 3 | 0.2 | -1.2 |
| CD93 | 8 | 0.8 | -2.3 |
| CD97 | 8 | 0.5 | -1.4 |
| CD4 | 1 | -0.9 | -1.0 |
| CD163 | 21 | 1.6 | -1.5 |
| CD36 | 5 | 0.4 | -3.7 |
| CD302 | 2 | 0.2 | -1.7 |
| CD52 | 0 | -0.6 | -2.5 |
| CD53 | 1 | -0.2 | -1.1 |
| CD34 | 5 | 0.7 | -1.7 |
| CD83 | 1 | -0.2 | -1.7 |
| CD37 | 0 | -1.4 | -1.3 |
| CD300LF | 2 | 0.0 | -2.2 |
| CD69 | 1 | -0.2 | -1.5 |
| CD101 | 3 | -1.1 | -2.8 |
| CD33 | 9 | 1.4 | -1.7 |
| CD300LG | 3 | 0.3 | -5.9 |
| CD300C | 4 | 0.8 | -2.3 |
| CD22 | 8 | 0.3 | -1.1 |
| CD274 | 3 | 0.4 | -1.2 |
| CD1C | 7 | 1.1 | -1.5 |
| CD79A | 1 | -0.1 | 2.0 |
| CD27 | 1 | -0.4 | 1.3 |
| CD244 | 7 | 1.0 | -1.4 |
| CD5L | 14 | 2.2 | -4.1 |
| CD300LB | 4 | 0.8 | -1.0 |
| CD160 | 1 | -0.1 | -1.0 |
| CD300E | 1 | -0.2 | -1.6 |
| CD19 | 3 | -0.1 | 2.5 |
| **Thrombospondin, type 1 repeat** | | | |
| ADAM metallopeptidase with thrombospondin type 1 motif 12 (ADAMTS12) | 62 | 4.4 | 1.2 |
| ADAM metallopeptidase with thrombospondin type 1 motif 14 (ADAMTS14) | 16 | 1.1 | 2.5 |
| ADAM metallopeptidase with thrombospondin type 1 motif 15 (ADAMTS15) | 12 | 0.8 | -2.2 |
| ADAM metallopeptidase with thrombospondin type 1 motif 16 (ADAMTS16) | 37 | 3.2 | 2.7 |
| ADAM metallopeptidase with thrombospondin type 1 motif 17 (ADAMTS17) | 15 | 1.5 | -1.0 |
| ADAM metallopeptidase with thrombospondin type 1 motif 18 (ADAMTS18) | 21 | 1.6 | 1.5 |
| ADAM metallopeptidase with thrombospondin type 1 motif 2 (ADAMTS2) | 24 | 2.4 | -0.1 |
| ADAM metallopeptidase with thrombospondin type 1 motif 20 (ADAMTS20) | 44 | 3.3 | 1.1 |
| ADAM metallopeptidase with thrombospondin type 1 motif 5 (ADAMTS5) | 23 | 2.5 | 0.0 |
| ADAM metallopeptidase with thrombospondin type 1 motif 8 (ADAMTS8) | 5 | 0.1 | -4.7 |
| ADAMTS Like 3 (ADAMTSL3) | 27 | 1.5 | -3.0 |
| ADAMTS Like 4 (ADAMTSL4) | 7 | -0.3 | -2.3 |
| adhesion G protein-coupled receptor B3(ADGRB3) | 46 | 3.4 | -2.6 |
| Adhesion G Protein-Coupled Receptor D1 (ADGRD1) | 14 | 1.3 | -2.4 |
| Adhesion G Protein-Coupled Receptor E1 (ADGRE1) | 14 | 1.2 | -3.2 |
| Adhesion G Protein-Coupled Receptor E3 (ADGRE3) | 6 | 0.2 | -3.4 |
| Adhesion G Protein-Coupled Receptor F5 (ADGRF5) | 7 | -0.7 | -2.1 |
| Adhesion G Protein-Coupled Receptor L2 (ADGRL2) | 22 | 1.4 | -2.1 |
| Adhesion G Protein-Coupled Receptor L3 (ADGRL3) | 40 | 3.8 | -2.1 |
| Adhesion G Protein-Coupled Receptor L4 (ADGRL4) | 27 | 3.0 | -1.3 |
| Adhesion G Protein-Coupled Receptor D1 (ADGRD1) | 14 | 1.3 | -2.4 |
| Alcohol Dehydrogenase 1A (Class I), Alpha Polypeptide (ADH1A) | 6 | 0.8 | -3.8 |
| Alcohol Dehydrogenase 1B (Class I), Beta Polypeptide (ADH1B) | 8 | 1.3 | -4.9 |
| Coagulation Factor X (F10) * | 3 | -0.1 | -2.1 |
| Coagulation Factor XI (F11) * | 12 | 1.3 | -5.0 |
| Coagulation Factor XII (F12) | 5 | 0.4 | 2.8 |
| semaphorin 5A (SEMA5A) * | 34 | 0.5 | -3.0 |
| semaphorin 6A (SEMA6A) * | 10 | -0.3 | -2.9 |
| semaphorin 6D (SEMA6D) * | 25 | 0.5 | -2.7 |
| thrombospondin type 1 domain containing 1 (THSD1) | 9 | 0.5 | -2.3 |
| thrombospondin type 1 domain containing 4 (THSD4) | 6 | -0.2 | -1.5 |
| thrombospondin type 1 domain containing 7A(THSD7A) | 32 | 2.2 | -1.1 |
| unc-5 netrin receptor D (UNC5D) | 30 | 2.9 | -0.9 |

**Table 3**. Genes associated with immune interactions and thrombosis. * Indicates gene in which expression change could be the result of intratumoral host cells.

| **Adaptations promoting malignant tumor growth** | | | | | |
| --- | --- | --- | --- | --- | --- |
| **Mitosis/Cell cycle** | | | | | |
| BR serine/threonine kinase 1 (BRSK1) | | 9 | 1.0 | 2.3 | |
| BUB1 mitotic checkpoint serine/threonine kinase B (BUB1B) | | 10 | 0.3 | 2.8 | |
| BUB1 mitotic checkpoint serine/threonine kinase (BUB1) | | 7 | -0.3 | 3.8 | |
| DLG associated protein 5 (DLGAP5) | | 4 | -0.5 | 4.1 | |
| E2F transcription factor 2 (E2F2) | | 3 | 0.1 | 2.5 | |
| E2F transcription factor 7 (E2F7) | | 12 | 0.8 | 2.5 | |
| E2F transcription factor 8 (E2F8) | | 2 | -1.3 | 3.6 | |
| ERCC excision repair 6 like, spindle assembly checkpoint helicase (ERCC6L) | | 2 | -1.8 | 3.0 | |
| FA complementation group I (FANCI) | | 5 | -1.0 | 2.1 | |
| G protein-coupled estrogen receptor 1 (GPER1) | | 0 | -1.7 | -2.6 | |
| Holliday junction recognition protein (HJURP) | | 7 | 0.3 | 3.9 | |
| NDC80 kinetochore complex component (NDC80) | | 4 | -0.2 | 3.5 | |
| NIMA related kinase 2 (NKD2) | | 0 | -1.8 | 3.8 | |
| NUF2 component of NDC80 kinetochore complex (NUF2) | | 9 | 1.2 | 4.1 | |
| Opa interacting protein 5 (OIP5) | | 1 | -0.3 | 2.6 | |
| PTTG1 regulator of sister chromatid separation, securing (PTTG1) | | 1 | -0.2 | 2.3 | |
| SPC25 component of NDC80 kinetochore complex (SPC25) | | 2 | 0.2 | 3.0 | |
| TPX2 microtubule nucleation factor (TPX2) | | 4 | -0.4 | 3.7 | |
| ZW10 interacting kinetochore protein (ZWINT) | | 10 | 1.8 | 2.7 | |
| anillin, actin binding protein (ANLN) | | 9 | 0.0 | 3.8 | |
| assembly factor for spindle microtubules (ASPM) | | 45 | 1.3 | 3.6 | |
| aurora kinase A (AURKA) | | 0 | -1.7 | 2.5 | |
| aurora kinase B (AURKB) | | 1 | -0.6 | 3.7 | |
| baculoviral IAP repeat containing 5 (BIRC5) | | 1 | 0.0 | 4.1 | |
| cell division cycle 20 (CDC20) | | 3 | -0.2 | 4.0 | |
| cell division cycle 25A (CDC25A) | | 3 | -0.1 | 2.5 | |
| cell division cycle 25C (CDC25C) | | 6 | 0.6 | 3.9 | |
| cell division cycle 45 (CDC45) | | 2 | -0.7 | 4.0 | |
| cell division cycle 6 (CDC6) | | 2 | -0.7 | 3.5 | |
| cell division cycle associated 2 (CDCA2) | | 5 | -0.6 | 3.1 | |
| cell division cycle associated 3 (CDCA3) | | 2 | 0.0 | 3.4 | |
| cell division cycle associated 5 (CDCA5) | | 1 | -0.3 | 3.0 | |
| cell division cycle associated 7 (CDCA7) | | 5 | 0.5 | 3.1 | |
| cell growth regulator with EF-hand domain 1(CGREF1) | | 3 | 0.3 | 3.4 | |
| centromere protein A (CENPA) | | 3 | 1.1 | 3.6 | |
| centromere protein E (CENPE) | | 20 | -0.2 | 2.7 | |
| centromere protein F (CENPF) | | 27 | 0.1 | 3.3 | |
| centromere protein I (CENPI) | | 5 | -0.2 | 3.1 | |
| centromere protein K (CENPK) | | 2 | 0.0 | 2.3 | |
| centromere protein M (CENPM) | | 0 | -1.1 | 2.1 | |
| centrosomal protein 55 (CEP55) | | 5 | 0.4 | 3.3 | |
| checkpoint kinase 1 (CHEK1) | | 3 | -0.1 | 2.1 | |
| chromatin assembly factor 1 subunit B (CHAF1B) | | 3 | -0.3 | 2.1 | |
| chromatin licensing and DNA replication factor 1(CDT1) | | 3 | 0.0 | 2.9 | |
| Claspin (CLSPN) | | 7 | -0.7 | 3.0 | |
| cyclin A2 (CCNA2) | | 3 | 0.0 | 2.9 | |
| cyclin B1 (CCNB1) | | 0 | -1.8 | 2.8 | |
| cyclin B2 (CCNB2) | | 0 | -1.7 | 3.4 | |
| cyclin E1 (CCNE1) | | 4 | 0.4 | 3.1 | |
| cyclin E2 (CCNE2) | | 1 | -0.8 | 2.2 | |
| cyclin F (CCNF) | | 3 | -0.7 | 2.0 | |
| cyclin O (CCNO) | | 2 | 0.3 | 2.5 | |
| cyclin dependent kinase 1 (CDK1) | | 1 | -0.5 | 2.3 | |
| Cyclin Dependent Kinase 5 Regulatory Subunit 2 (CDK5R2) | | 0 | -1.7 | 2.1 | |
| cyclin dependent kinase inhibitor 2A (CDKN2A) | | 17 | 3.7 | 2.3 | |
| cyclin dependent kinase inhibitor 3 (CDKN3) | | 0 | -1.2 | 3.2 | |
| dual specificity phosphatase 1 (DUSP1) | | 0 | -1.7 | -2.0 | |
| dual specificity phosphatase 13 (DUSP13) | | 1 | -0.4 | 3.0 | |
| epithelial cell transforming 2 (ECT2) | | 6 | -0.2 | 2.0 | |
| Epithelial Cell Transforming 2 Like 2 (ECT2L) | | 7 | 0.0 | -2.2 | |
| establishment of sister chromatid cohesion N-acetyltransferase 2 (ESCO2) | | 3 | -0.4 | 2.7 | |
| family with sequence similarity 107 member A (FAM107A) | | 1 | 0.0 | -4.9 | |
| family with sequence similarity 83 member A (FAM83A) | | 2 | -0.3 | 6.0 | |
| family with sequence similarity 83 member D (FAM83D) | | 5 | 0.4 | 2.3 | |
| forkhead box M1 (FOXM1) | | 6 | 0.0 | 3.4 | |
| helicase, lymphoid specific (HELLS) | | 2 | -1.2 | 2.9 | |
| high mobility group AT-hook 1 (HMGA1) | | 0 | -0.8 | 2.1 | |
| high mobility group AT-hook 2 (HMGA2) | | 3 | 1.6 | 2.9 | |
| kallikrein related peptidase 10 (KLK10) | | 2 | 0.3 | -2.5 | |
| kallikrein related peptidase 11 (KLK11) | | 3 | 0.6 | -2.8 | |
| kinesin family member 1A (KIF1A) | | 21 | 1.6 | 2.3 | |
| kinesin family member 11 (KIF11) | | 4 | -0.9 | 2.8 | |
| kinesin family member 14 (KIF14) | | 5 | -1.5 | 3.7 | |
| kinesin family member 15 (KIF15) | | 5 | -1.1 | 3.2 | |
| kinesin family member 17 (KIF17) | | 12 | 0.8 | -2.2 | |
| kinesin family member 18B (KIF18B) | | 6 | 0.2 | 3.8 | |
| kinesin family member 19 (KIF19) | | 17 | 2.1 | -2.0 | |
| kinesin family member 20A (KIF20A) | | 3 | -0.9 | 3.5 | |
| kinesin family member 23 (KIF23) | | 3 | -1.0 | 2.7 | |
| kinesin family member 2C (KIF2C) | | 6 | 0.1 | 4.0 | |
| kinesin family member 4A(KIF4A) | | 8 | -0.2 | 4.2 | |
| kinesin family member 6 (KIF6) | | 9 | 0.6 | -2.0 | |
| Kinesin Family Member C1 (KIFC1) | | 7 | 0.4 | 2.8 | |
| marker of proliferation Ki-67 (MKI67) | | 17 | -1.1 | 3.0 | |
| maternal embryonic leucine zipper kinase (MELK) | | 1 | -1.3 | 4.0 | |
| minichromosome maintenance complex component 2 (MCM2) | | 4 | -0.6 | 2.0 | |
| minichromosome maintenance complex component 4 (MCM4) | | 6 | -0.1 | 2.1 | |
| minichromosome maintenance complex component 10 (MCM10) | | 10 | 0.6 | 3.6 | |
| mitogen-activated protein kinase 4 (MAPK4) | | 6 | 0.7 | -4.1 | |
| mitotic arrest deficient 2 like 1 (MAD2L1) | | 2 | 0.2 | 2.5 | |
| neural precursor cell expressed, developmentally down-regulated 9 (NEDD9) | | 0 | -2.6 | -2.0 | |
| non-SMC condensin I complex subunit G (NCAPG) | | 4 | -0.8 | 3.4 | |
| non-SMC condensin I complex subunit H (NCAPH) | | 6 | 0.1 | 3.3 | |
| nucleolar and spindle associated protein 1 (NUSAP1) | | 4 | 0.6 | 2.7 | |
| polo like kinase 1 (PLK1) | | 5 | 0.2 | 3.5 | |
| polo like kinase 4 (PLK4) | | 8 | 0.1 | 2.0 | |
| protein kinase C epsilon (PRKCE) | | 6 | 0.1 | -2.1 | |
| protein kinase, membrane associated tyrosine/threonine 1 (PKMYT1) | | 2 | -0.2 | 3.3 | |
| protein regulator of cytokinesis 1 (PRC1) | | 2 | -0.8 | 2.5 | |
| regulator of cell cycle (RGCC) | | 1 | 0.5 | -3.0 | |
| regulator of chromosome condensation 1 (RCC1) | | 1 | -0.9 | 2.1 | |
| sperm associated antigen 4 (SPAG4) | | 0 | -1.8 | 3.0 | |
| sperm associated antigen 5 (SPAG5) | | 5 | -0.9 | 3.3 | |
| sperm associated antigen 6 (SPAG6) | | 9 | 1.1 | -2.6 | |
| spindle and kinetochore associated complex subunit 1 (SKA1) | | 1 | -0.4 | 3.0 | |
| spindle and kinetochore associated complex subunit 3 (SKA3) | | 2 | -0.3 | 3.3 | |
| structural maintenance of chromosomes 1B (SMC1B) | | 7 | -0.5 | 2.5 | |
| tubulin polymerization promoting protein (TPPP) | | 1 | -0.2 | -2.3 | |
| tubulin polymerization promoting protein 3(TPPP3) | | 0 | -1.1 | -3.0 | |
| ubiquitin conjugating enzyme E2C (UBE2C) | | 2 | 0.5 | 4.1 | |
| Ubiquitin Conjugating Enzyme E2T (UBE2T) | | 2 | 0.2 | 3.4 | |
| **Transcription regulation** | | | | | |
| MAX network transcriptional repressor (MNT) | 0 | | -2.1 | | 0.2 |
| MLLT1 super elongation complex subunit (MLLT1) | 0 | | -2.1 | | -0.5 |
| POU class 3 homeobox 4 (POU3F2) | 4 | | 0.8 | | 2.0 |
| POU class 2 homeobox 4 (POU2AF1) | 3 | | 0.5 | | 2.0 |
| SATB homeobox 2 (SATB2) | 20 | | 2.4 | | 0.9 |
| SWI/SNF related, matrix associated, actin dependent regulator of chromatin, subfamily a, member 4 (SMARCA4) | 35 | | 2.9 | | 0.3 |
| T-box transcription factor 3 (TBX3) | 8 | |  | | -2.3 |
| T-box transcription factor 4 (TBX4) | 12 | |  | | -2.8 |
| T-box transcription factor 5 (TBX5) | 14 | |  | | -2.0 |
| T-box transcription factor 15 (TBX15) | 16 | | 2.2 | | 2.7 |
| TATA-box binding protein associated factor 1 like (TAF1L) | 41 | | 2.6 | | -0.1 |
| TATA-box binding protein associated factor 6 like (TAF6L) | 0 | | -2.2 | | 0.3 |
| TOX high mobility group box family member 4 (TOX4) | 0 | | -2.2 | | 0.0 |
| Zic family member 1 (ZIC1) | 21 | | 2.9 | | 1.3 |
| calmodulin binding transcription activator 2 (CAMTA2) | 1 | | -2.2 | | -0.5 |
| clock circadian regulator (CLOCK) | 0 | | -2.6 | | 0.2 |
| cyclin K (CCNK) | 0 | | -2.1 | | 0.2 |
| cysteine and serine rich nuclear protein 1 (CSRNP1) | 0 | | -2.2 | | -2.4 |
| dachshund family transcription factor 1 (DACH1) | 16 | | 2.2 | | -3.1 |
| dachshund family transcription factor 2 (DACH2) | 21 | | 2.9 | | -0.5 |
| forkhead box F1 (FOXF1) | 4 | | 1.0 | | -2.9 |
| forkhead box F2 (FOXF2) | 8 | | 2.2 | | -1.7 |
| heat shock transcription factor 1 (HSF1) | 0 | | -2.0 | | 0.5 |
| homeobox A1(HOXA1) | 15 | | 2.4 | | 1.0 |
| homeobox A10 (HOXA10) | 1 | | -2.6 | | 2.4 |
| homeobox A3 (HOXA3) | 14 | | 2.3 | | -0.2 |
| homeobox A4 (HOXA4) | 4 | | 1.4 | | -2.0 |
| homeobox A5(HOXA5) | 11 | | 2.1 | | -2.0 |
| homeobox B9 (HOXB9) | 2 | | 0.3 | | 3.1 |
| homeobox C10 (HOXC10) | 4 | | 0.5 | | 2.7 |
| homeobox C11 (HOXC11) | 6 | | 1.2 | | 2.5 |
| homeobox C13 (HOXC13) | 5 | | 1.5 | | 2.8 |
| hypoxia inducible factor 1 subunit alpha (HIF1A) | 0 | | -2.6 | | 0.6 |
| Endothelial PAS Domain Protein 1 (EPAS1) (also HIF2A) | 9 | | 0.5 | | -2.7 |
| hypoxia inducible factor 3 subunit alpha (HIF3A) | 8 | | 0.8 | | -2.3 |
| inhibin subunit beta A (INHBA) | 15 | | 2.2 | | -1.0 |
| interferon regulatory factor 2 binding protein 1 (IRF2BP1) | 0 | | -2.1 | | 0.0 |
| interferon regulatory factor 5 (IRF5) | 0 | | -2.0 | | -0.2 |
| interferon regulatory factor2BP1 (IRF2BP1) | 0 | | -2.1 | | 0.0 |
| interferon regulatory factor 3(IRF3) | 0 | | -2.0 | | 0.6 |
| iroquois homeobox 1 (IRX1) | 11 | | 2.3 | | -3.6 |
| iroquois homeobox 2 (IRX2) | 11 | | 2.4 | | -2.3 |
| iroquois homeobox 6 (IRX6) | 5 | | 0.5 | | -2.1 |
| mesenchyme homeobox 2 (MEOX2) | 11 | | 2.3 | | -1.6 |
| Musculin (MSC) | 8 | | 2.1 | | 0.9 |
| nucleus accumbens associated 1 (NACC1) | 0 | | -2.0 | | 0.7 |
| paired box 7 (PAX7) | 5 | | 0.4 | | 2.1 |
| paternally expressed 3 (PEG3) | 49 | | 3.5 | | -1.9 |
| protein kinase C beta (PRKCB) | 19 | | 2.2 | | -1.0 |
| Protein Kinase C Epsilon (PRKCE) | 6 | | 0.1 | | -2.1 |
| Protein Kinase C Theta (PRKCQ) | 5 | | -0.1 | | -2.0 |
| Protein Kinase CGMP-Dependent 1 (PRKG1) | 13 | | 1.4 | | -2.1 |
| Protein Kinase CGMP-Dependent 2 (PRKG2) | 7 | | 0.2 | | -3.5 |
| retinoid X receptor beta (RXRB) | 0 | | -2.0 | | 0.1 |
| spalt like transcription factor 1 (SALL1) | 30 | | 2.3 | | 1.8 |
| spalt like transcription factor 4 (SALL4) | 17 | | 1.3 | | 2.9 |
| sterol regulatory element binding transcription factor 2 (SREBF2) | 1 | | -2.0 | | -0.7 |
| Splicing Regulatory Glutamic Acid and Lysine Rich Protein 1 (SREK1) | 0 | | -2.2 | | 0.2 |
| storkhead box 2 (STOX2) | 0 | | -2.8 | | -0.5 |
| teashirt zinc finger homeobox 2 (TSHZ2) | 25 | | 2.2 | | 0.0 |
| teashirt zinc finger homeobox 3 (TSHZ3) | 41 | | 3.6 | | -0.6 |
| transcription factor AP-2 delta (TFAP2D) | 6 | | 0.8 | | 3.7 |
| transcriptional repressor GATA binding 1 (TRPS1) | 35 | | 2.8 | | 0.3 |
| tumor protein p53 (TP53) | 177 | | 11.9 | | 0.4 |
| upstream binding protein 1 (UBP1) | 0 | | -2.1 | | -0.4 |
| vestigial like family member 3 (VGLL3) | 14 | | 2.3 | | -1.8 |
| VGLL4 | 0 | | -1.5 | | 0.1 |
| zinc finger E-box binding homeobox 1 (ZEB1) | 26 | | 2.2 | | -1.5 |
| zinc finger and BTB domain containing 1 (ZBTB1) | 0 | | -2.4 | | -0.1 |
| zinc finger homeobox 4 (ZFHX4) | 104 | | 5.5 | | 0.8 |
| zinc finger protein 205 (ZNF205) | 0 | | -2.1 | | 0.4 |
| zinc finger protein 208 (ZNF208) | 35 | | 2.8 | | 0.1 |
| zinc finger protein 257 (ZNF257) | 18 | | 2.3 | | 0.3 |
| zinc finger protein 26 (ZNF26) | 0 | | -2.1 | | 0.6 |
| zinc finger protein 30 (ZNF30) | 0 | | -2.2 | | 0.2 |
| zinc finger protein 324 (ZNF324) | 0 | | -2.1 | | -0.3 |
| zinc finger protein 433 (ZNF433) | 0 | | -2.3 | | -0.2 |
| zinc finger protein 521 (ZNF521) | 30 | | 2.3 | | -1.0 |
| zinc finger protein 57 (ZNF57) | 0 | | -2.1 | | 0.0 |
| zinc finger protein 648 (ZNF648) | 14 | | 2.2 | | 0.8 |
| zinc finger protein 655 (ZNF655) | 0 | | -2.0 | | -0.1 |
| zinc finger protein 676 (ZNF676) | 27 | | 3.2 | | -0.2 |
| zinc finger protein 749 (ZNF749) | 0 | | -2.5 | | 0.4 |
| zinc finger protein 84 (ZNF84) | 0 | | -2.4 | | 0.2 |
| zinc finger protein 846 (ZNF846) | 0 | | -2.0 | | -0.3 |
| zinc finger protein 98 (ZNF98) | 16 | | 2.0 | | 0.4 |
| zinc finger protein, FOG family member 2 (ZFPM2) | 36 | | 3.1 | | -1.3 |
| **Circadian entrainment** | | | | | |
| ADCYAP receptor type I (ADCYAP1R1) | 14 | | 2.0 | | -1.5 |
| GNAS complex locus (GNAS) | 16 | | 2.0 | | 0.4 |
| adenylate cyclase 2 (ADCY2) | 29 | | 2.6 | | -0.9 |
| adenylate cyclase 8 (ADCY8) | 29 | | 2.6 | | -4.5 |
| calcium voltage-gated channel subunit alpha1 C (CACNA1C) | 33 | | 2.1 | | -1.1 |
| glutamate ionotropic receptor AMPA type subunit 4 (GRIA4) | 22 | | 2.1 | | 0.3 |
| glutamate ionotropic receptor NMDA type subunit 2A (GRIN2A) | 32 | | 2.3 | | -0.7 |
| glutamate ionotropic receptor NMDA type subunit 3A (GRIN3A) | 27 | | 2.6 | | -0.3 |
| guanylate cyclase 1 soluble subunit alpha 2 (GUCY1A2) | 19 | | 2.2 | | -1.8 |
| phospholipase C beta 1(PLCB1) | 34 | | 2.8 | | 0.3 |
| Potassium Inwardly Rectifying Channel Subfamily J Member 15 (KCNJ15) | 3 | | 0.1 | | -2.6 |
| Potassium Inwardly Rectifying Channel Subfamily J Member 16 (KCNJ16) | 7 | | 0.9 | | -2.2 |
| potassium inwardly rectifying channel subfamily J member 3 (KCNJ3) | 23 | | 3.0 | | -0.6 |
| potassium inwardly rectifying channel subfamily J member 5 (KCNJ5) | 3 | | 0.0 | | -2.4 |
| protein kinase C beta (PRKCB) | 19 | | 2.2 | | -1.0 |
| protein kinase C gamma (PRKCG) | 16 | | 2.1 | | 1.3 |
| Protein Kinase C Theta (PRKCQ) | 5 | | -0.1 | | -2.0 |
| ryanodine receptor 1 (RYR1) | 56 | | 1.5 | | -0.5 |
| ryanodine receptor 2(RYR2) | 125 | | 5.6 | | -1.8 |
| ryanodine receptor 3(RYR3) | 66 | | 2.2 | | -0.9 |
| clock circadian regulator (CLOCK) | 0 | | -2.6 | | 0.2 |
| **RNA Polymerase II** | | | | | |
| MAX network transcriptional repressor (MNT) | 0 | | -2.1 | | 0.2 |
| MLLT1 super elongation complex subunit (MLLT1) | 0 | | -2.1 | | -0.5 |
| SATB homeobox 2 (SATB2) | 20 | | 2.4 | |  |
| SWI/SNF related, matrix associated, actin dependent regulator of chromatin, subfamily a, member 4 (SMARCA4) | 35 | | 2.9 | | 0.3 |
| T-box transcription factor 15 (TBX15) | 16 | | 2.2 | | 2.7 |
| T-box transcription factor 18 (TBX18) | 9 | | 1.2 | | 1.2 |
| T-box transcription factor 15 (TBX21) | 10 | | 1.8 | | -1.0 |
| T-box transcription factor 15 (TBX3) | 8 | | 1.5 | | -2.3 |
| T-box transcription factor 15 (TBX4) | 12 | | 1.7 | | -2.8 |
| T-box transcription factor 15 (TBX5) | 14 | | 1.9 | | -2.0 |
| TATA-box binding protein associated factor 1 like (TAF1L) | 41 | | 2.6 | | -0.1 |
| TATA-box binding protein associated factor 6 like (TAF6L) | 0 | | -2.2 | | 0.3 |
| TOX high mobility group box family member 4 (TOX4) | 0 | | -2.2 | | 0.0 |
| Zic family member 1 (ZIC1) | 21 | | 2.9 | | 1.3 |
| calmodulin binding transcription activator 2 (CAMTA2) | 1 | | -2.2 | | -0.5 |
| clock circadian regulator (CLOCK) | 0 | | -2.6 | | 0.2 |
| cyclin K (CCNK) | 0 | | -2.1 | | 0.2 |
| cysteine and serine rich nuclear protein 1 (CSRNP1) | 0 | | -2.2 | | -2.4 |
| dachshund family transcription factor 1 (DACH1) | 16 | | 2.2 | | -3.1 |
| dachshund family transcription factor 2 (DACH2) | 21 | | 2.9 | | -0.5 |
| forkhead box F1 (FOXF1) | 4 | | 1.0 | | -2.9 |
| forkhead box F2 (FOXF2) | 8 | | 2.2 | | -1.7 |
| Forkhead Box M1 (FOXM1) | 6 | | 0.0 | | 3.4 |
| Forkhead Box P3 (FOXP3) | 1 | | -0.6 | | 2.2 |
| heat shock transcription factor 1 (HSF1) | 0 | | -2.0 | | 0.5 |
| homeobox A1 (HOXA1) | 15 | | 2.4 | | 1.0 |
| homeobox A3 (HOXA3) | 14 | | 2.3 | | -0.2 |
| homeobox A5 (HOXA5) | 11 | | 2.1 | | -2.0 |
| homeobox B13 (HOXB13) | 3 | | 0.4 | | 2.2 |
| homeobox B9 (HOXB9) | 2 | | 0.3 | | 3.1 |
| homeobox C10 (HOXC10) | 4 | | 0.5 | | 2.7 |
| homeobox C11 (HOXC11) | 6 | | 1.2 | | 2.5 |
| homeobox C13 (HOXC13) | 5 | | 1.5 | | 2.8 |
| hypoxia inducible factor 1 subunit alpha (HIF1A) | 0 | | -2.6 | | 0.6 |
| Inhibin Subunit Alpha (INHA) | 3 | | 0.3 | | 2.3 |
| inhibin subunit beta A (INHBA) | 15 | | 2.2 | | -1.0 |
| interferon regulatory factor 2 binding protein 1(IRF2BP1) | 0 | | -2.1 | | 0.0 |
| interferon regulatory factor 3 (IRF3) | 0 | | -2.0 | | 0.6 |
| interferon regulatory factor 5 (IRF5) | 0 | | -2.0 | | -0.2 |
| iroquois homeobox 1 (IRX1) | 11 | | 2.3 | | -3.6 |
| iroquois homeobox 2 (IRX2) | 11 | | 2.4 | | -2.3 |
| iroquois homeobox 6 (IRX6) | 5 | | 0.5 | | -2.1 |
| mesenchyme homeobox 2 (MEOX2) | 11 | | 2.3 | | -1.6 |
| Musculin (MSC) | 8 | | 2.1 | | 0.9 |
| nucleus accumbens associated 1 (NACC1) | 0 | | -2.0 | | 0.7 |
| paternally expressed 3 (PEG3) | 49 | | 3.5 | | -1.9 |
| protein kinase C beta (PRKCB) | 19 | | 2.2 | | -1.0 |
| protein kinase C epsilon (PRKCE) | 6 | | 0.1 | | -2.1 |
| protein kinase C theta (PRKCQ) | 5 | | -0.1 | | -2.0 |
| retinoid X receptor beta (RXRB) | 0 | | -2.0 | | 0.1 |
| retinoid X receptor gamma (RXRG) | 13 | | 1.8 | | -4.1 |
| spalt like transcription factor 1 (SALL1) | 30 | | 2.3 | | 1.8 |
| spalt like transcription factor 4 (SALL4) | 17 | | 1.3 | | 2.9 |
| sterol regulatory element binding transcription factor 2 (SREBF2) | 1 | | -2.0 | | -0.7 |
| storkhead box 2 (STOX2) | 0 | | -2.8 | | -0.5 |
| teashirt zinc finger homeobox 2 (TSHZ2) | 25 | | 2.2 | | 0.0 |
| teashirt zinc finger homeobox 3 (TSHZ3) | 41 | | 3.6 | | -0.6 |
| transcription factor AP-2 delta (TFAP2D) | 6 | | 0.8 | | 3.7 |
| transcriptional repressor GATA binding 1 (TRPS1) | 35 | | 2.8 | | 0.3 |
| tumor protein p53 (TP53) | 177 | | 11.9 | | 0.4 |
| upstream binding protein 1 (UBP1) | 0 | | -2.1 | | -0.4 |
| vestigial like family member 3 (VGLL3) | 14 | | 2.3 | | -1.8 |
| zinc finger E-box binding homeobox 1 (ZEB1) | 26 | | 2.2 | | -1.5 |
| zinc finger and BTB domain containing 1 (ZBTB1) | 0 | | -2.4 | | -0.1 |
| zinc finger homeobox 4 (ZFHX4) | 104 | | 5.5 | | 0.8 |
| zinc finger protein 205 (ZNF205) | 0 | | -2.1 | | 0.4 |
| zinc finger protein 208 (ZNF208) | 35 | | 2.8 | | 0.1 |
| zinc finger protein 257 (ZNF257) | 18 | | 2.3 | | -0.3 |
| zinc finger protein 26 (ZNF26) | 0 | | -2.1 | | 1.5 |
| zinc finger protein 30 (ZNF30) | 0 | | -2.2 | | 0.2 |
| zinc finger protein 324 (ZNF324) | 0 | | -2.1 | | -0.3 |
| zinc finger protein 433 (ZNF433) | 0 | | -2.3 | | -0.2 |
| zinc finger protein 521 (ZNF521) | 30 | | 2.3 | | -1.0 |
| zinc finger protein 57 (ZNF57) | 0 | | -2.1 | | 0.0 |
| zinc finger protein 648 (ZNF648) | 14 | | 2.2 | | 0.8 |
| zinc finger protein 655 (ZNF655) | 0 | | -2.0 | | 0.2 |
| zinc finger protein 676 (ZNF676) | 27 | | 3.2 | | -0.2 |
| zinc finger protein 749 (ZNF749) | 0 | | -2.5 | | 0.4 |
| zinc finger protein 846 (ZNF846) | 0 | | -2.0 | | -0.3 |
| zinc finger protein 98 (ZNF98) | 16 | | 2.0 | | 0.4 |
| zinc finger protein, FOG family member 2 (ZFPM2) | 36 | | 3.1 | | -1.3 |
| mRNA editing | | | | | |
| Apolipoprotein B MRNA Editing Enzyme Catalytic Subunit 1 (APOBEC1) | 1 | | -0.3 | | 1.5 |
| Apolipoprotein B MRNA Editing Enzyme Catalytic Subunit 3A (APOBEC3A) | 3 | | 0.7 | | -1.1 |
| Apolipoprotein B MRNA Editing Enzyme Catalytic Subunit 3B (APOBEC3B) | 0 | | -1.7 | | 2.1 |
| Apolipoprotein B MRNA Editing Enzyme Catalytic Subunit 4 (APOBEC4) | 3 | | 0.2 | | -2.7 |

**Supplemental Table 4.** Strong promotion of signaling pathways not associated with normal lung function provide pro-proliferative signaling and promote phenotypic plasticity for optimal fitness of cancer cells growing in spatially and temporally heterogeneous environments.

| Canonical signaling pathways | | | |
| --- | --- | --- | --- |
| Gene | Number of samples mutated (n=313) | Standard deviation of distance from neutral line | Expression change compared to normal lung (base 2) |
| MAPK Pathways | | | |
| MAP2K1 | 7 | 1.1 | 0.0 |
| MAP2K3 | 7 | 1.1 | -0.3 |
| MAP2K4 | 4 | 0.4 | -0.2 |
| MAP2K5 | 2 | -0.3 | -0.4 |
| MAP2K6 | 4 | 0.5 | 0.7 |
| MAP2K7 | 4 | 0.6 | -0.1 |
| MAP3K1 | 1 | -2.5 | 0.0 |
| MAP3K10 | 2 | -1.0 | 0.3 |
| MAP3K11 | 2 | -1.1 | -0.2 |
| MAP3K12 | 3 | -0.9 | -0.4 |
| MAP3K13 | 5 | -0.5 | 0.5 |
| MAP3K15 | 9 | 0.2 | -2.7 |
| MAP3K2 | 3 | -0.1 | 0.2 |
| MAP3K3 | 5 | 0.0 | -1.1 |
| MAP3K4 | 9 | -0.6 | -0.6 |
| MAP3K5 | 5 | -1.1 | -0.3 |
| MAP3K6 | 2 | -1.8 | -0.4 |
| MAP3K7 | 2 | -0.8 | 0.2 |
| MAP3K8 | 2 | -0.5 | -1.1 |
| MAP3K9 | 14 | 1.1 | 0.5 |
| MAP4K1 | 7 | 0.4 | 0.0 |
| MAP4K2 | 5 | 0.0 | -0.3 |
| MAP4K3 | 8 | 0.2 | 0.2 |
| MAP4K4 | 6 | -0.4 | 0.1 |
| MAP4K5 | 0 | -2.6 | 0.0 |
| MAPK1 | 2 | -0.1 | -0.2 |
| MAPK10 | 3 | -0.1 | -2.0 |
| MAPK11 | 1 | -0.4 | -0.6 |
| MAPK12 | 1 | -0.5 | 0.5 |
| MAPK13 | 0 | -1.7 | 0.6 |
| MAPK14 | 3 | 0.2 | 0.0 |
| MAPK15 | 2 | -0.6 | -0.6 |
| MAPK1IP1L | 2 | 0.1 | -0.2 |
| MAPK3 | 3 | 0.2 | -0.4 |
| MAPK4 | 6 | 0.7 | -4.1 |
| MAPK6 | 4 | -0.3 | 0.5 |
| MAPK7 | 4 | -0.3 | -0.2 |
| MAPK8 | 3 | 0.0 | 0.2 |
| MAPK8IP1 | 3 | -0.2 | -0.1 |
| MAPK8IP3 | 7 | -0.5 | 1.0 |
| MAPK9 | 4 | 0.3 | 0.0 |
| MAPKAP1 | 3 | -0.2 | 0.0 |
| MAPKAPK2 | 2 | -0.3 | 0.1 |
| MAPKAPK3 | 1 | -0.7 | -0.3 |
| MAPKAPK5 | 4 | 0.5 | 0.4 |
| MAPKBP1 | 6 | -1.0 | -0.5 |
| EGF | 10 | 0.1 | 1.7 |
| ERAS | 2 | 0.4 | 0.5 |
| HRAS | 2 | 0.3 | 0.1 |
| NRAS | 4 | 0.9 | 0.2 |
| P53 pathway | | | |
| GTSE1 | 2 | -1.0 | 3.3 |
| CHEK1 | 3 | -0.1 | 2.1 |
| CHEK2 | 6 | 0.5 | 1.5 |
| CCNB1 | 0 | -1.8 | 2.8 |
| CCNB1IP1 | 3 | 0.4 | -0.1 |
| CCNB2 | 0 | -1.7 | 3.4 |
| CCNB3 | 20 | -1.8 | -0.8 |
| CCNE1 | 4 | 0.4 | 3.1 |
| CCNE2 | 1 | -0.8 | 2.2 |
| CCNF | 3 | -0.4 | 2.0 |
| CDK1 | 1 | -0.5 | 2.3 |
| CDKN2A | 17 | 3.7 | 2.3 |
| IGFBP1 | 4 | 1.2 | 0.7 |
| IGFBP2 | 2 | 0.4 | 0.5 |
| IGFBP3 | 0 | -1.5 | 2.1 |
| IGFBP4 | 1 | 0.0 | -0.6 |
| IGFBP5 | 1 | -0.1 | 0.3 |
| RRM1 | 7 | 0.2 | 0.7 |
| RRM2 | 2 | -0.2 | 3.2 |
| SERPINB4 | 14 | 2.1 | 1.5 |
| SERPINB5 | 3 | 0.1 | 2.5 |
| ERBB Pathways | | | |
| ERBB2 | 9 | -0.1 | 0.7 |
| ERBB2IP | 5 | -1.1 | 0.0 |
| ERBB3 | 9 | -0.2 | 0.5 |
| ERBB4 | 25 | 1.8 | -3.3 |
| WNT pathway | | | |
| WNT1 | 6 | 1.5 | 0.1 |
| WNT10A | 7 | 1.5 | 1.2 |
| WNT10B | 6 | 1.0 | 0.9 |
| WNT11 | 3 | 0.6 | -1.7 |
| WNT16 | 6 | 0.9 | 0.8 |
| WNT2 | 4 | 0.4 | -1.4 |
| WNT2B | 3 | 0.2 | -1.6 |
| WNT3 | 0 | -1.6 | 1.7 |
| WNT3A | 6 | 1.1 | -5.0 |
| WNT4 | 1 | -0.6 | -0.7 |
| WNT5A | 1 | -0.5 | -0.3 |
| WNT5B | 3 | 0.2 | 0.4 |
| WNT7A | 11 | 1.8 | -4.1 |
| WNT7B | 3 | 0.2 | 0.3 |
| WNT8B | 5 | 0.7 | 0.5 |
| WNT9A | 3 | 0.4 | -1.6 |
| WNT9B | 3 | 0.3 | -0.7 |
| Transforming Growth Factor | | | |
| TGFA | 0 | -1.0 | 0.7 |
| TGFB1 | 2 | 0.1 | -0.6 |
| TGFB1I1 | 4 | 0.3 | -0.9 |
| TGFB2 | 8 | 1.1 | -1.5 |
| TGFB3 | 0 | -1.8 | 0.0 |
| TGFBI | 5 | 0.1 | 0.2 |
| TGFBR1 | 5 | 0.4 | -0.2 |
| TGFBR2 | 2 | -0.7 | -1.9 |
| TGFBR3 | 15 | 1.4 | -3.0 |
| TGFBRAP1 | 8 | 0.3 | -0.1 |
| AKT Pathways | | | |
| AKT1 | 2 | -0.5 | -0.1 |
| AKT2 | 6 | 0.6 | 0.2 |
| AKT3 | 6 | 0.6 | -1.3 |
| AKTIP | 0 | -1.5 | -0.4 |
| PTEN | 6 | 0.8 | -0.4 |
| NOTCH Pathway | | | |
| NOTCH1 | 12 | -0.7 | -1.0 |
| NOTCH2 | 15 | -0.7 | -0.1 |
| NOTCH2NL | 5 | 1.0 | 0.1 |
| NOTCH3 | 9 | -0.6 | 0.7 |
| NOTCH4 | 32 | 1.8 | -1.9 |
| SMAD pathway | | | |
| SMAD1 | 4 | 0.2 | 0.4 |
| SMAD2 | 8 | 1.0 | -0.3 |
| SMAD3 | 5 | 0.6 | 0.0 |
| SMAD4 | 12 | 1.5 | -0.5 |
| SMAD7 | 0 | -1.8 | -1.2 |
| SMAD9 | 3 | 0.0 | -2.3 |
| PIK3 Pathway | | | |
| PIK3AP1 | 9 | 0.6 | -0.9 |
| PIK3C2A | 10 | -0.6 | -0.5 |
| PIK3C2B | 16 | 0.4 | -0.4 |
| PIK3C2G | 13 | 0.5 | -0.4 |
| PIK3C3 | 11 | 0.7 | -0.2 |
| PIK3CA | 15 | 1.0 | -0.2 |
| PIK3CB | 9 | 0.1 | 0.1 |
| PIK3CD | 3 | -1.1 | -0.2 |
| PIK3CG | 24 | 2.0 | -0.9 |
| PIK3R1 | 3 | -0.6 | -1.2 |
| PIK3R2 | 0 | -2.4 | 0.5 |
| PIK3R3 | 4 | 0.2 | -0.8 |
| PIK3R4 | 14 | 0.5 | -0.1 |
| PIK3R5 | 6 | 0.0 | -1.3 |
| RB/MYC pathways | | | |
| RB1 | 19 | 1.9 | -0.4 |
| MYC | 2 | -0.5 | -0.2 |
| MYCBP | 0 | -0.8 | 0.3 |
| MYCBP2 | 26 | -1.2 | -0.4 |
| MYCBPAP | 3 | -1.0 | -0.6 |
| HIPPO pathway | | | |
| YAP1 | 0 | -2.0 | -0.3 |
| TAZ | 2 | 0.1 | 0.5 |
| MST1 | 2 | -1.0 | 1.1 |
| LATS1 | 13 | 0.7 | -0.3 |
| LATS2 | 7 | 0.0 | -1.2 |
| TEAD1 | 2 | -0.4 | -0.5 |
| TEAD3 | 3 | 0.1 | 0.4 |
| TEAD4 | 3 | 0.0 | -0.4 |

Table 6. Canonical signaling pathways

1. Fumagalli S, Ivanenkov VV, Teng T, Thomas G. Suprainduction of p53 by disruption of 40S and 60S ribosome biogenesis leads to the activation of a novel G2/M checkpoint. Genes Dev. 2012;26(10):1028-40.

2. Bursac S, Brdovcak MC, Pfannkuchen M, Orsolic I, Golomb L, Zhu Y, et al. Mutual protection of ribosomal proteins L5 and L11 from degradation is essential for p53 activation upon ribosomal biogenesis stress. Proc Natl Acad Sci U S A. 2012;109(50):20467-72.

3. Dai MS, Arnold H, Sun XX, Sears R, Lu H. Inhibition of c-Myc activity by ribosomal protein L11. EMBO J. 2007;26(14):3332-45.

4. Zhang H, Liu J, Dang Q, Wang X, Chen J, Lin X, et al. Ribosomal protein RPL5 regulates colon cancer cell proliferation and migration through MAPK/ERK signaling pathway. BMC Mol Cell Biol. 2022;23(1):48.

5. Jiang L, Li T, Zhang X, Zhang B, Yu C, Li Y, et al. RPL10L Is Required for Male Meiotic Division by Compensating for RPL10 during Meiotic Sex Chromosome Inactivation in Mice. Curr Biol. 2017;27(10):1498-505 e6.
